# A network approach to analyze neuronal lineage and layer innervation in the *Drosophila* optic lobes

**DOI:** 10.1371/journal.pone.0227897

**Published:** 2020-02-05

**Authors:** Alberto del Valle Rodríguez, Martín Cera, José R. Portillo

**Affiliations:** 1 Center for Genomics and Systems Biology, New York University Abu Dhabi (NYUAD), Abu Dhabi, UAE; 2 Departamento de Matemática Aplicada I, Universidad de Sevilla, Sevilla, Spain; 3 Instituto Universitario de Investigación de Matemáticas de la Universidad de Sevilla (IMUS), Sevilla, Spain; University of Mississippi, UNITED STATES

## Abstract

The optic lobes of the fruit fly *Drosophila melanogaster* form a highly wired neural network composed of roughly 130.000 neurons of more than 80 different types. How neuronal diversity arises from very few cell progenitors is a central question in developmental neurobiology. We use the optic lobe of the fruit fly as a paradigm to understand how neuroblasts, the neural stem cells, generate multiple neuron types. Although the development of the fly brain has been the subject of extensive research, very little is known about the lineage relationships of the cell types forming the adult optic lobes. Here we perform a large-scale lineage bioinformatics analysis using the graph theory. We generated a large collection of cell clones that genetically label the progeny of neuroblasts and built a database to draw graphs showing the lineage relationships between cell types. By establishing biological criteria that measures the strength of the neuronal relationships and applying community detection tools we have identified eight clusters of neurons. Each cluster contains different cell types that we pose are the product of eight distinct classes of neuroblasts. Three of these clusters match the available lineage data, supporting the predictive value of the analysis. Finally, we show that the neuronal progeny of a neuroblast do not have preferential innervation patterns, but instead become part of different layers and neuropils. Here we establish a new methodology that helps understanding the logic of *Drosophila* brain development and can be applied to the more complex vertebrate brains.

## Introduction

Over a century ago Cajal and Sanchez initiated studies on the neuroanatomy of the insect brains [1]. Their Golgi impregnations from flies, bees or horseflies provided the first insights on the neural circuits and neuron types present in their exquisitely organized brains. Later work by Fischbach and Dittrich in *Drosophila* using the same technique provided a comprehensive catalogue of the multiple cell types populating the optic lobe (OL) [2]. In recent years, the *Drosophila* nervous system has been studied with extraordinary precision up to the single cell level thanks to the development of modern clonal analysis techniques (reviewed in [3] and [4]) combined with the availability of thousands of gene/cell specific lines [5], [6] and the use of high-resolution electron microscopy [7], [8].

Despite its small size the fly brain is capable of accomplishing a variety of complex behaviors. The optic lobes in both sides of the central brain account for over 80% of the total neurons of the brain, whose activity result in motion detection [9, 10], the processing of color vision [11], [12] or polarized light detection [13]. This versatility is achieved by the precisely assembled neuronal circuits arising from the well-diversified collection of neuron types of the optic lobes [2, 14].

**Fig 1:**
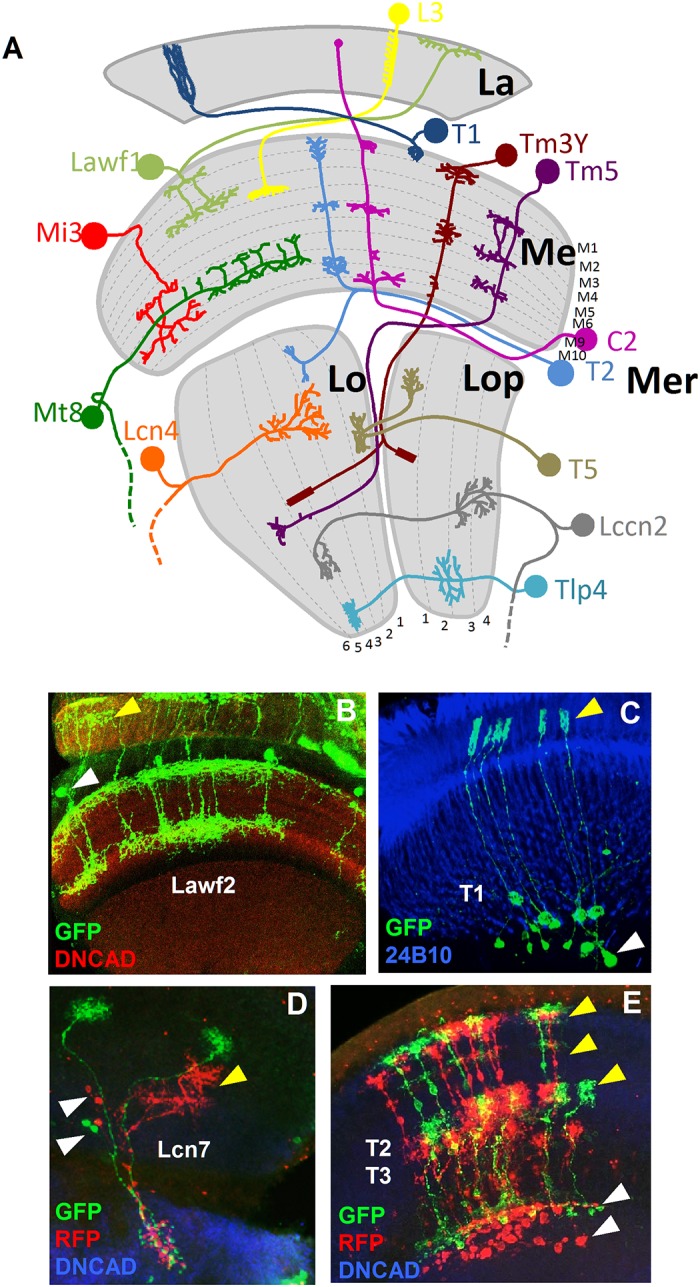
Neuron types in the optic lobe. (A) Model showing a sample of neuron types in the optic lobe. The neuropils of the OL and the layers of each neuropil are shown in grey. La: lamina. Me: medulla. Lo: lobula. Lop: lobula plate. Mer: medulla rim. (B) Lawf2 neurons homogeneous MARCM clone. Arrowheads indicate projections in the lamina (yellow) and cell bodies in the medulla cortex (white). Projections reach M1 and M9 of the medulla neuropil (labeled with DN-cadherin in red). (C) T1 homogeneous MARCM clone. Arrowheads indicate projection in the lamina (yellow), cell bodies in the medulla cortex (white). (D) Lcn7 neurons homogeneous twin spot MARCM clone. Cell bodies (white arrowheads) and projections (yellow arrowheads) remain in the lobula. (E) T2-T3 neurons homogeneous twin spot MARCM clone. Projections are in the medulla (white arrowhead) and lobula (yellow arrowhead). (Neuropils in D and E stained in blue with DN-cadherin).

Each optic lobe has approximately 60,000 neurons of over 80 neuron types. They are distributed in four retinotopically-organized ganglia that lay beneath the retina: the lamina, the medulla, the lobula and the lobula plate (see Fig 1A). The photoreceptors (PR) from the retina receive the light input and innervate the lamina and the medulla, where they synapse with other neurons. The lamina is composed by ~4,000 neurons of 6 cell types (the monopolar L1 to L5 neurons and a lamina intrinsic Lai amacrine cell). This relatively simple structure contrasts with the medulla, composed by ~40,000 neurons of over 70 cell types. Their projections in the medulla result in 10 synaptic layers (M1 to M10) [2, 15]. The majority of the medulla cells have their cell bodies in the medulla cortex, between the medulla and the lamina neuropils. Local neurons project only in the medulla and include intrinsic, distal and proximal medulla neurons (Mi, Dm and Pm respectively). In contrast, projecting neurons arborize into deeper layers interconnecting the medulla with the lobula (Tm transmedullary neurons) or with the medulla, the lobula and the lobula plate (TmY neurons). The medulla tangential neurons (Mt), with a descending axon towards the central brain, project widely in parallel to several layers of the medulla.

The remaining ~15,000 neurons belong to the lobula complex, with two separate neuropils orthogonal to the medulla: the lobula and the lobula plate, with 6 and 4 synaptic layers respectively. Four cell types form a crescent that surrounds part of the proximal medulla, with cell bodies in the medulla rim. These neurons innervate the medulla and lamina centrifugally (C2 and C3) or bifurcate in a “T” shape with an ascending axon to the medulla and a descending axon towards the lobula (T2 and T3). T4 and T5 cell bodies sit below these neurons and interconnect the lobula plate with the proximal medulla (T4 cells) or with the lobula (T5 cells). Translobula plate neurons (Tlp), with soma in the proximal lobula cortex, interconnect the lobula and the lobula plate. Additionally, Y cells bifurcate in the inner chiasma to project in the proximal medulla and the lobula. Another two major classes of neurons reach the central brain: the lobula columnar neurons, (Lcn) that innervate different layers of the lobula and the central brain (CB), and the lobula complex columnar neurons (Lccn), which connect the lobula plate and the lobula with the CB. Finally, two classes of giant tangential neurons innervate the lobula plate and include the horizontal system cells (HS) and vertical system cells (VS).

Recent studies have shown how the neuroblasts (NBs) of the OL proliferate and diversify during larval stages. The generation of neuronal diversity involves the spatial and temporal activation of transcription factors during neurogenesis that determine the fate of the lineage of a NB [16–18]. Exhaustive screenings using combinations of antibodies that label multiple transcription factors (TFs) helped understanding the temporal sequence of genes expressed in NBs during larval stages and their early cell lineages. However, very little is known about the lineage relationships of the multiple adult neuron types that they produce. Besides, it is not possible to decipher the lineage of the adult OL cells using this approach because in most cases TF expression in NBs is not maintained in the adult neurons.

The development of the lineage tracing techniques MARCM and Twin Spot MARCM have greatly contributed to lineage studies of the *Drosophila* nervous system [19, 20]. These techniques allow the labeling of the progeny of a NB by inducing the expression of fluorescent proteins that label their daughter cells. The expression of fluorophores in the adult neurons reveals clonally related populations. The analysis of cell type relationships based on clonal analysis together with the use of tissue-specific drivers helped understanding the development and lineage relationships of mushroom body neurons [21], the olfactory system [22] or regions of the ventral nerve cord [23]. However the use of tissue-specific drivers may result in the loss of partial lineages because some neurons may not express it. To overcome this limitation, in an effort to find the lineage relationships of as many cell types as possible of the OL, we used ubiquitous neuronal drivers. The expression of these markers is maintained along development and remains in the adult neurons. We generated a database with our collection of clones and used tools from the graph theory, a mathematical approach capable of processing large and complex data sets, with the aim of clustering these neuron types into lineage groups. Graphs represent collections of objects (nodes) that are linked by edges when there is a defined relation between them. The graph theory has proven a powerful tool for addressing diverse problems, ranging from genome and protein organization, [24], [25] prediction of protein function [26] or population genetics [27]. In Neuroscience it has facilitated the understanding of the *C*. *elegans* connectome [28] or the functional organization of the human brain [29]. Here we propose a novel use of the graph theory to establish a methodology that assesses lineage relationships between neuron types of the OL of *Drosophila*. We combined the current knowledge on how neuroblasts divide to generate neuron diversity, with intensive data computing to find clusters of neuron types sharing a common precursor and to analyze their connectivity.

## Results

### Generating cell clones to analyze cell lineage in the optic lobes: Clones selection and classification

To study the lineage of the wide variety of cell types within the OL we performed a large-scale clonal analysis by using the twin spot MARCM system [20], which allows marking the daughter neurons of a dividing NB. In the nervous system of *Drosophila*, a NB generally divides asymmetrically to self-renew and produce a ganglion mother cell (GMC) that divides once more into two neurons (S1A Fig). This process is repeated multiple times, so that a NB may sequentially express different transcription factors leading to different cell types [16]. With the twin spot MARCM, the release of a molecular repressor by a heat-shock allows the clonally inherited expression of one of two fluorescent markers (GFP or RFP) in each daughter cell of a dividing precursor (a GMC or a NB). The number and distribution of the marked cells varies depending on the stage and type of precursor where the recombination event took place (S1A Fig). We also included some samples with the MARCM system [19] (less than 5% of the clones) that follows a very similar approach to the Twin Spot MARCM but labels neurons only with GFP [19]. To prevent the loss of partial lineages, we used ubiquitous drivers (*actin5c*, *tubulin* and *elav*) that label the entire progeny of a NB. Because chances of heat shocking a single or very few NBs per OL are very low, many of our clones had a large number of cells (>100) with overlapping axons and cell bodies that made neuron type identification impossible. Consequently from the approximately initial 7500 clones we selected for our analysis 350, where the morphological identification of neurons was possible. We classified each clone considering the neuron types marked with GFP and/or RFP as well as the quality, size, symmetry, homogeneity and ability to recognize the neuron types within the clone (S1 Table).

Most of the clones analyzed contained multiple cell types. Although it might be safe to assume that neurons within a clone of few cells (20 neurons or less) are related by lineage, the nature of our analysis does not exclude the possibility of overlapping clones generated by the final divisions of two or more NBs. Rather than considering all the cell types within a clone as part of the same progeny, we studied pairs of cell types as independent events, accounting for the repetition of pairs of neuron types in our clone collection. Although most of the clones analyzed contained multiple cell types, the simplest exhibited only one cell type (homogeneous cell clones), indicating that at least the last rounds of divisions of the NB generated the same cell type. We recently reported this mode of division for the Lawf1 and Lawf2 neurons [30] (Fig 1B). We show a similar pattern for the neuron types T1 (Fig 1C) or Lcn4 (Fig 3C). Our results also indicate that NBs generating T2-T3 neurons may divide symmetrically as it can be inferred from the two resulting populations, with similar number of RFP and GFP labeled neurons (Fig 1E). A similar case is shown for the Lcn7 neurons (Fig 1D). Some of our clones only show one of the GFP or RFP markers, indicating that cell death plays an important role during the neurogenesis of the OL (S1 B), in agreement with the apoptosis reported at larval stages [16–18].

### Using the graph theory to analyze cell lineage in the OL

To study lineage relationships of the OL neuron types with a higher throughput we used a network approach. This approach allows us to assess the strength of the relationships between pairs of neurons in our dataset and cluster those that are closely related and therefore share a common NB.

**Fig 2:**
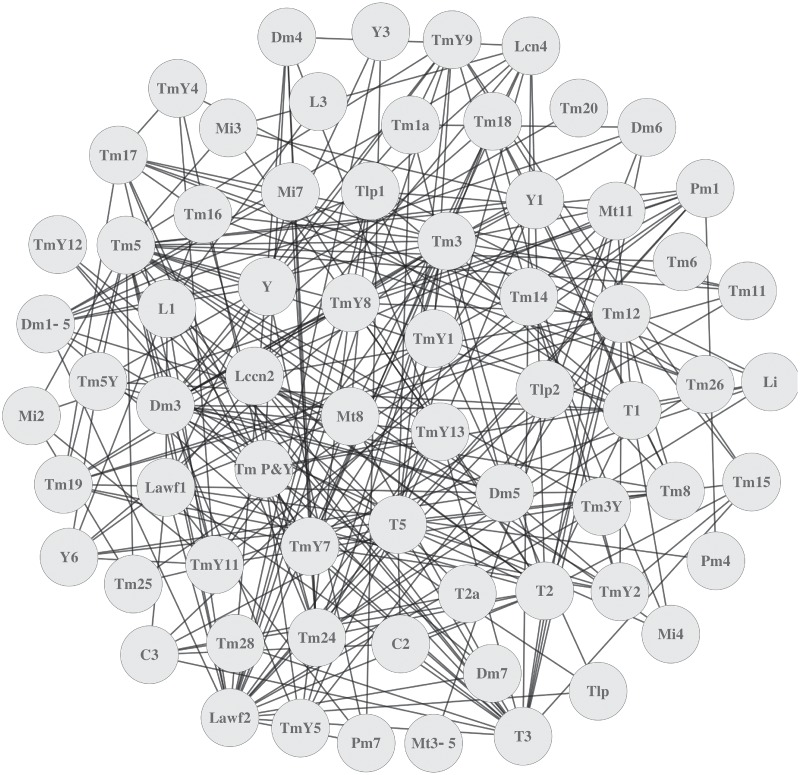
Graph representing the neuron types in the clone collection. Each node of the graph represents one of the 68 neuron types found in our clones. Two nodes are linked by an edge if the corresponding pair of neurons appears in one clone.

Neuronal diversity within a clone arises from two different sources. First, a NB can generate different neuron types following a temporal sequence, so the number of cell types depends on the stage where the NB was labeled. Second, one clone can be the product of more than one NB and, as a result, large clones exhibit wide combinations of cell types. To study the lineage relationships between these types, we represented the neurons of our clones as a network of interacting nodes. Although two cell types in a clone are not necessarily related by lineage, if the same neuron pair appears in multiple clones, the likelihood of the relationship increases. The graph theory provides the tools to formally describe and analyze a network. It provides a representation of the objects or nodes forming the network and the interactions between them. A graph is defined as a pair (V,E) where V is a set of objects and E is a subset of V representing the relationships or edges between them. Thus, two nodes i and j from *V* are adjacent if the pair (i,j) belongs to E. We computed the relationships between neuron types of all our clones (S1 Table) to build a graph where neurons are vertices linked by an edge whenever they appear together in at least one clone (Fig 2 and S2 Fig). However neurons that appear together in these graphs do not necessarily share the same precursor. The quantity and quality of the information varies from clone to clone and depends on multiple parameters. For instance, whereas homogeneous small cell clones clearly indicate a common NB, large clones with several cell types labeled by both GFP and RFP may arguably be the product of more than one NB. In the latter case, the resulting neuron types may or may not be related by lineage. To assess this, we provide a measure of the quality of the information by setting a scoring system that weighs the relative contribution of each clone in the evaluation of pairwise relationships. The neuron types, the total number of neurons within the clone or the colors labeling them are all parameters that can be used to calculate the strength of this relationship. We scored our clones (0 to 1) according to these variables so higher values (e.g. >0.75) indicate stronger relationships between two cell types. We defined this score as the *reliability* (R) of the relationship between two neuron types, adding a weigh that assess the lineage connection between vertices.

The first parameter to measure the R is the quality (q) of the GFP signal in the clone and ranges from 0 to 4 (S2 Table). A clear GFP signal, where all the neuron processes were identified leads to the highest score (4), whereas clones where processes of some neurons are lost or intermingled have a low q value (0–1). The q is a useful parameter to discard low quality clones or for comparative analysis between lower versus higher quality clones. We represent each of our clones in the form:

q(A(g,r),B(g,r))

**Fig 3:**
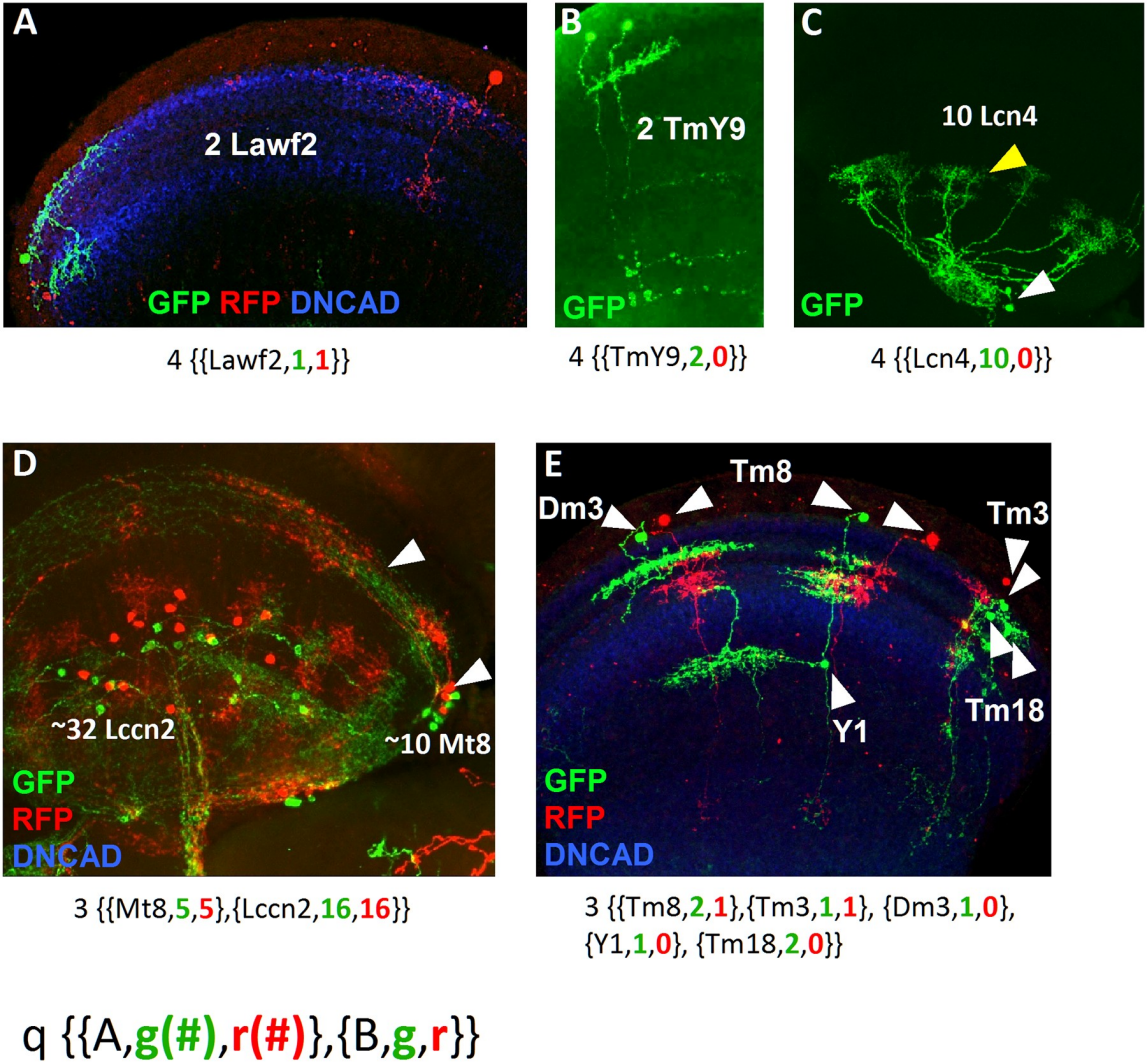
Samples summarizing the types of clones. (A) Sister cell clones of Lawf2 labeled with GFP and RFP projecting in the medulla neuropil (blue). (B) Sister cell clone of TmY9 neurons labeled by GFP projecting in the medulla and the lobula neuropils. (C) Homogeneous clone of 10 Lcn4 lobula neurons, with cell bodies (white arrowheads) and projections (yellow arrowheads) in the lobula. (D) Cell clone showing two different neuron types: the lobula Lccn2 and the medulla Mt8 (white arrowheads point cell bodies in the medulla rim and projections in the medulla), both labeled by GFP and RFP. (E) Cell clone with 5 different medulla neuron types (Dm3, Tm8, Y1, Tm3 and Tm18) combining uni-columnar, multi-columnar, local and projecting neurons labeled by GFP and RFP. The nomenclature for each clone is indicated below. Medulla and lobula neuropils stained in blue with DN-cadherin.

Where A and B are a pair of neuron types, g is the number of cells labeled by GFP and r is the number of cells labeled by RFP. Fig 3 shows some samples of the clones, ranging from the simplest ones, which are the product of the division of a GMC (Fig 3A and 3B) or the last rounds of division of a NB (Fig 3C) to complex ones, representing a symmetric division of the NB and the generation of 2 neuron types (Fig 3D) or multiple neuron types that might be the product of several NBs (Fig 3E).

The second parameter for measuring R is the total number of neurons in a clone. We defined all the possible combinations of pairs of neuron types within the clones and scored them following a biological rationale (see clones scoring method in Material and methods). Subsequently we calculated R for every pair of neuron types within each clone (see an example in S2 Fig). We integrated this information in the graphs from Fig 2 to generate weighted graphs (*V*,*E*) where the weigh of the edges is given by R. If *i* and *j* are two vertices (i.e. neuron types) from V, for each clone k where *i* and *j* are present simultaneously, we denote *r*_*ij*_^*k*^ as the R of the relationship between two different neuron types *i* and *j*. Each pair of *i* and *j* is an independent event. To measure the total reliability *r*_*ij*_ of two neuron types we use the principle of inclusion-exclusion, similar to the union of probabilistic independent events (see Methods). The *r*_*ij*_ values for all the neuron pairs provide the edges to build new weighted graphs (S4A and S4B Fig).

**Fig 4:**
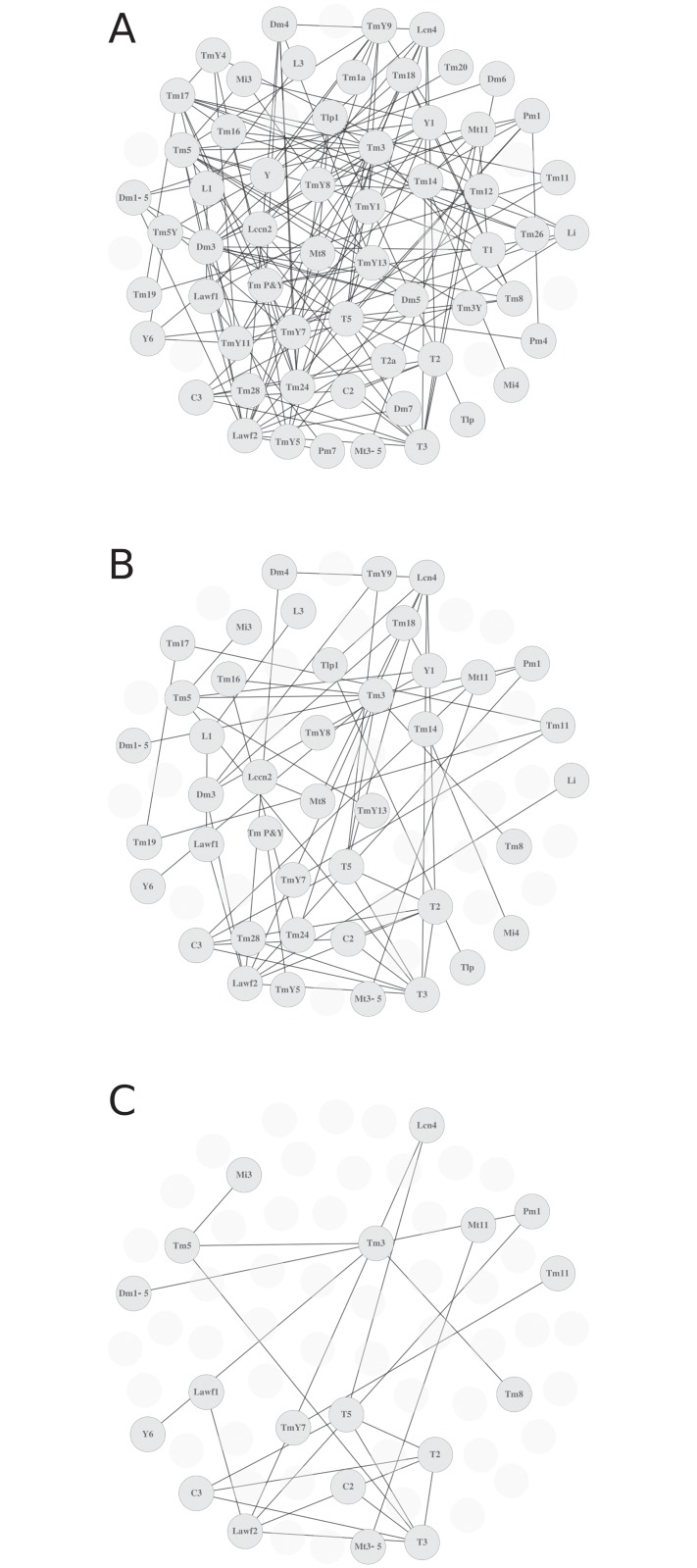
Discrete graphs for two different R values. (A) Graph built using an R value of 0.5. (B) and (C) show increasing R values (0.75 and 0.9), resulting in strength of the nodes relationships, but also in loss of nodes with lower representation in our dataset.

We built graphs for R values of 0.5, 0.75 and 0.95 (Fig 4A–4C) as well as 0 and 0.95 (S5A and S5B Fig). By increasing the R threshold, we increase the strength of the relationship of the nodes displayed. In this case, while enhancing the strength of the relationship, we also lose cell types with lower representation in our sample. We aim to find an optimal R with a strong correlation between pairs while minimizing the loss of neuron types. But most importantly, we aim to understand lineage relationships between groups of cell types rather than pairs of neurons.

### Community detection techniques to find lineage-related neuron types

To approach the broader question of which cell types share a common progenitor, we attempted a community detection analysis. Biological networks commonly present high concentration of edges between groups of vertices that in turn, display low connectivity with other vertices. These groups of nodes are called communities. For instance, in graphs representing the protein interactome, communities represent clusters of functionally related proteins [26, 31]. For our purpose, an edge with a high weight (R) between two vertices indicates a strong lineage relationship. We identified clusters of neurons with high edge concentration that must arguably correspond to the progeny of the same NB. From the different tools for community detection assayed we opted for the optimal modularity algorithm (*Q*) because it allows defining community partitions within a graph and provide a measure of the goodness of the partition (see Methods).

**Fig 5:**
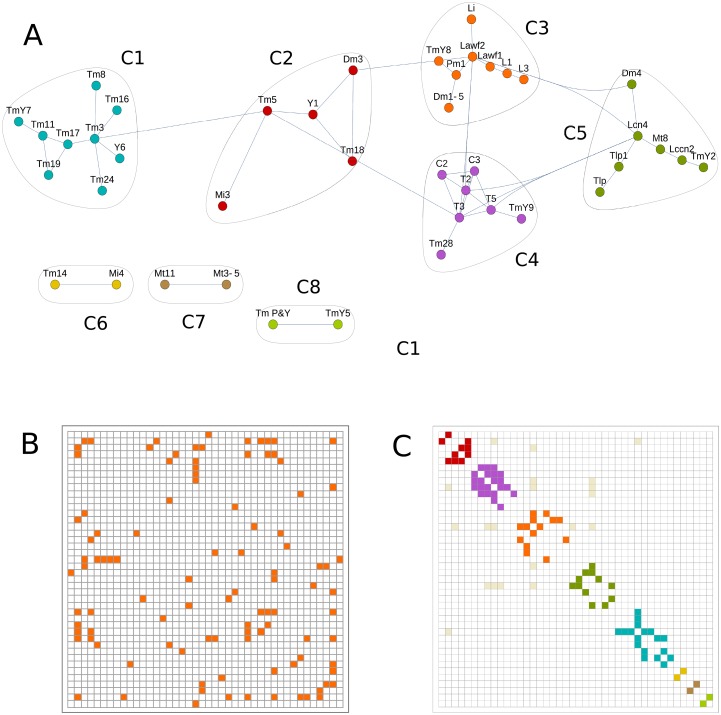
Community detection techniques find clusters of neurons related by lineage. (A) Community detection graph for R≥0.75 and clones with quality of 2–4. This graph represents the optimal modularity value and identifies 8 communities (C1 to C8) of neuron types with tightly connected nodes, indicating that each group may be the product of one class of NB. (B) Adjacency matrix from the previous graph indicating the connected nodes. This matrix can be arranged in communities by adding a color code (C).

We built graphs from clones with q values 2–4 and R intervals between 0 and 0.95 to compare the resulting community structures (Fig 5A, and S6 Fig). Our graphs analyses show inter-community edges that may be interpreted as neurons that belong to two different communities. We exclude these relationships because the same neuron type cannot be generated by two different NBs. In fact the density of inter-community edges is low and can be explained by random repetition of pairs of neurons in clones generated by more than one NB. We also represent the weighted graphs in adjacency matrices (Fig 5B) that show neuron types sharing an edge in the graph (an adjacency matrix for all our clones is shown in S7A Fig). These matrices provide information about the graph structure and can also be arranged in communities (Fig 5C and S7B Fig). As shown for the weighted graphs, we also lose neuron types in the community graphs as we increase R. For instance, R≥0.5 shows 57 neuron types divided in 4 communities (S6C Fig) whereas the community graph with clones with R equal or greater than 0.75 contains 42 neuron types forming 8 different communities (Fig 5A). As we increase R, although the content of the communities is almost identical, their number is reduced due to the loss of neuron types.

**Fig 6:**
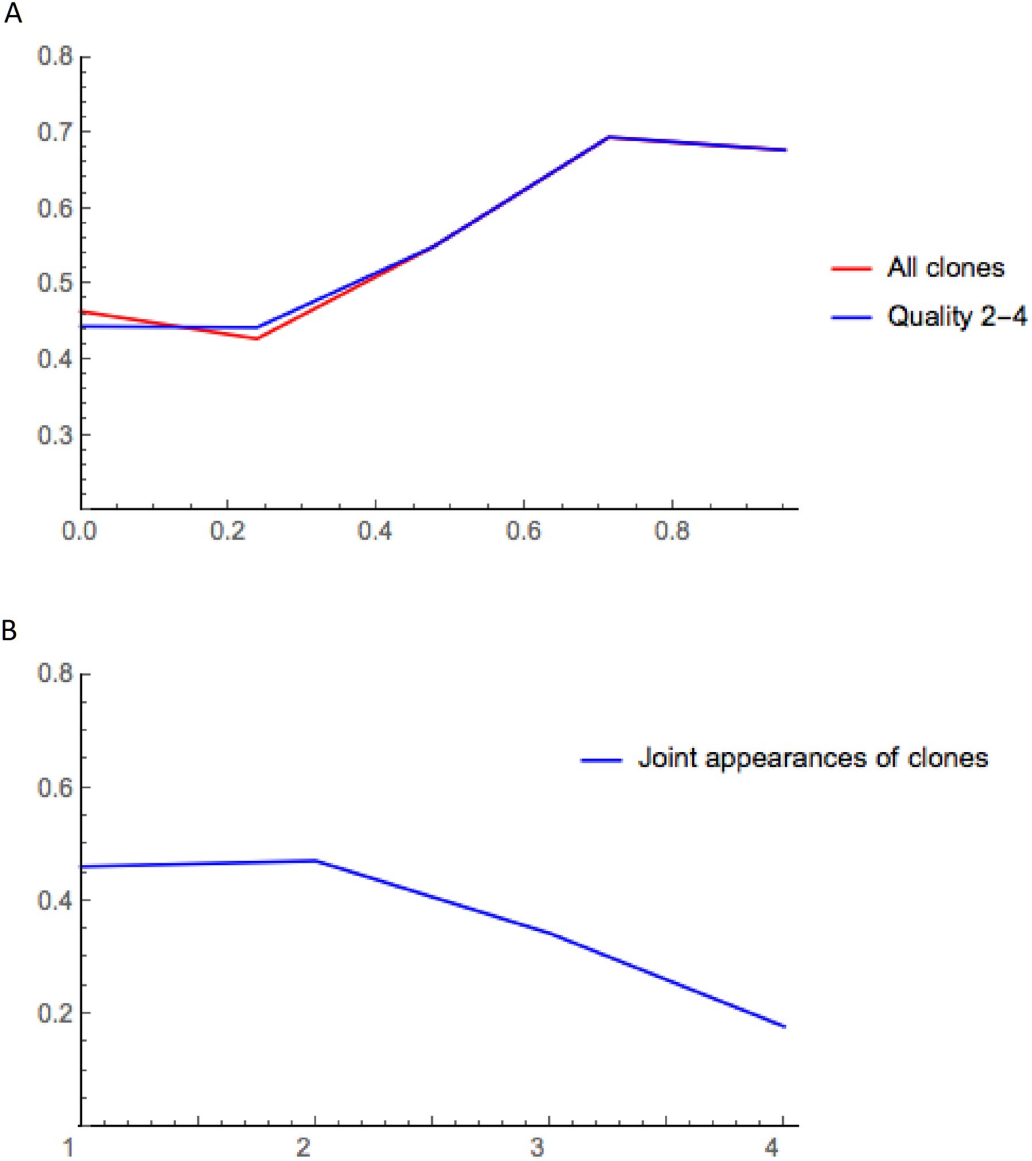
Representation of modularity (Q) as a function of the reliability (R) and the occurrence. (A) The modularity of the communities grows as R increases, reaching a maximum for R≥0.75. We show the modularity for the communities that include clones of any quality (red) versus clones of higher quality (2–4, blue). (B) Modularity of graphs as a function of the occurrence. The modularity decays with the increase of the edges due to the drastic reduction of the size sample.

To find the best community match we use modularity (Q), a measure that estimates the goodness of the partition of a network. In fact the modularity indicates whether or not a partition has community structure. The maximum value (Q = 1) indicates perfect community structure, and while values equal or lower than 0.3 indicate bad community structure, negative values indicate that the graph has no community structure [32]. We calculated the modularity for communities with R-values of 0 to 0.95 (Fig 6A) using all the clones with all qualities and clones with quality 2–4. In both cases the modularity first improves with higher thresholds, and maximum modularity is observed for R 0.75, matching the notion that modularity grows with the number of edges in the graph despite the reduction in the number of vertices and edges [33]. The modularity is not affected by the quality of the clones because low reliability clones disappear as we increase the threshold.

We analyzed in more detail the community graph with maximum modularity (R≥0.75). High modularity values imply a stronger relationship between the neurons in a cluster. This supports the idea that the neuron types within a cluster arise from the same NB, so we posit that the 8 communities of this graph are generated by 8 distinct classes of NBs. Although communities C6, C7 and C8 are too small and most likely incomplete, communities C1 to C5 range from 5 to 10 neuron types. A close look to the morphologies of cell types within these communities indicates that unlike the central brain, where NBs commonly produce similar cell types projecting to only a few different neuropils [34, 35], the NBs of the OL produce neurons with non-related morphologies projecting to sparse layers of different neuropils. For instance, the projecting columnar medulla neuron Tm5 and the local non-columnar lobula neuron Y1 belong to the community C2. Similar instances are found in communities C1 (neurons Tm3 and Y6, R = 0.960379) or C6 (Tm14 and Mi4, R = 0.855). However, community C1 shows almost exclusively Tm type neurons, which share similar shape. In the OL, 800 medulla columns act as functional units matching the 800 ommatidia, with R7 and R8 photoreceptors innervating the medulla to replicate a retinotopic map in the brain. Each unit is innervated by 800 uni-columnar (UC) neurons, with projections restricted to one column, and multi-columnar (MC) neurons, with wider projections that include several columns and are less numerous [7]. A recent work [18] shows that whereas UC neurons come from all NBs in the outer proliferative center of the medulla (OPC), the MC neurons are born from NBs that are in spatially delimited regions. Interestingly, 7 out of the 9 neuron types in community C1 are MC (all except Tm1 and Tm3). We propose that a regionalized NB produce these cell types, so the cell fate switches after each round of the NB division.

The NBs producing community C2 follow a similar strategy, with 4 MC neurons and 1 UC neuron. In fact all the communities have predominantly MC cell types, except the community C6, which may correspond to a non-regionalized NB producing Tm14 and Mi4 UC neurons. In summary, the NBs producing these communities (except for community C6) may belong to spatially restricted areas with distinct gene expression patterns that determine the cell types that they generate.

It is remarkable that the communities we found match the lineage data available so far in the OL. Previous lineage studies relating Mt8, Lccn2 and Lcn neurons are coincident with the types in the community C5; Lawf1 and Lawf2 neurons are part of the community C3 [30]; and T2, T3 and T5 neurons are part of the community C4 [36] (see Fig 5A and Discussion). All these observations support the idea that the 8 communities we define are the product of 8 distinct classes of NBs.

To compare the effect of the biological criteria (R) *versus* graphs representing the occurrence (number of events where two neuron types are in the same clone), we built graphs using the occurrence. The vertices of occurrence graphs share an edge when two neurons appear together, so the weight corresponds to the number of times two neuron types appear in a clone (S8 Fig). We applied community detection to these graphs and calculated their modularity (Fig 6B). In these conditions the modularity values are lower, and they decrease as the weight of the edges increases. This indicates that occurrence alone is a poor tool to establish lineage relationships between neuron types, at least in a sample limited by size. Here we show that the information included in R significantly improves the quality of the communities. We conclude that the use of R is critical to analyze the cell lineage in complex systems where there is an intrinsic size constraint or whenever only an approximation is sought.

**Fig 7:**
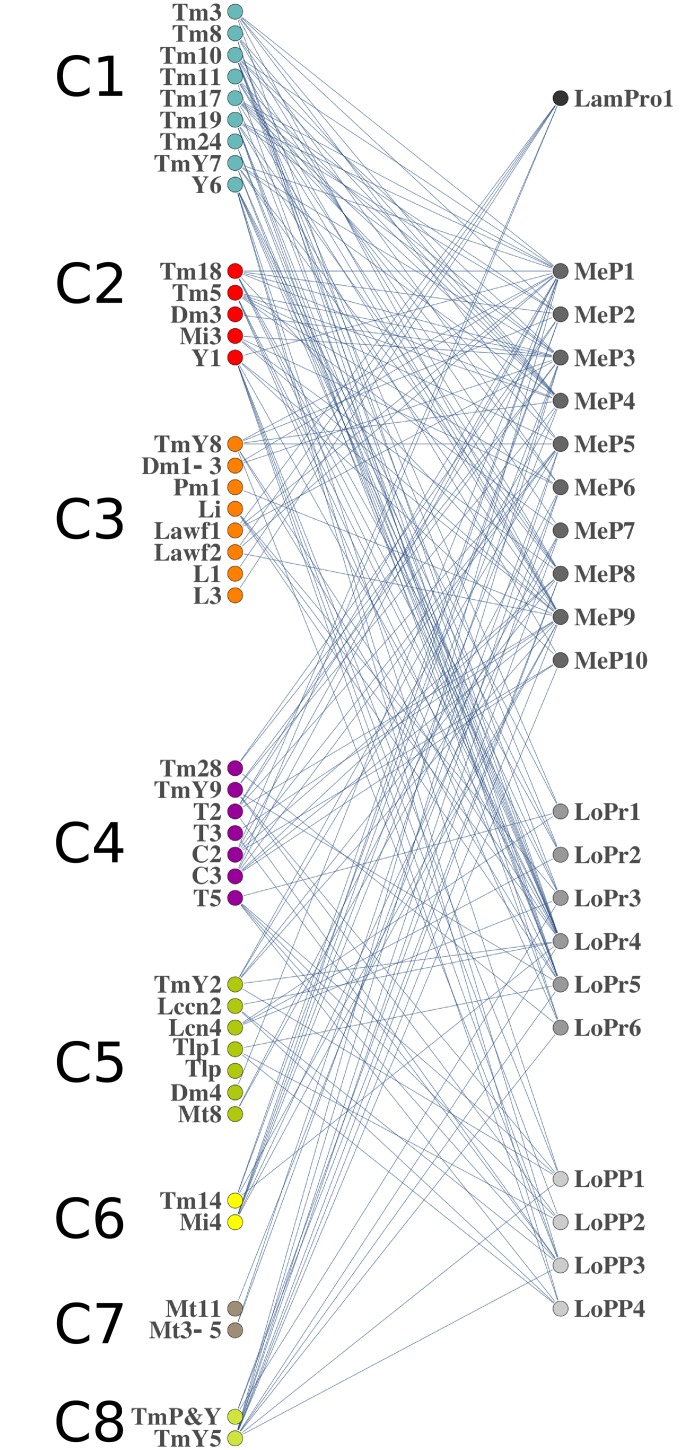
Projections of the neuron types in the different communities. The graph shows the projections of the neuron types in each community (C1 to C8) to the layers 1, 1–10, 1–6, 1–4 of the lamina, medulla, lobula and lobula plate neuropils, respectively. The communities show a heterogeneous projection patter, indicating that communities do not have an apparent neuropil or layer projection preference.

We finally pondered whether neuron types of the same community innervate the same neuropil or the same layers within a neuropil. We analyzed the neuronal arborizations to different layers of the OL for the neurons of the 8 communities (S9 Fig). Our data shows a heterogeneous distribution of the projections, which indicates that NBs of the OL generate neurons with no neuropil or layer preference. Additionally, we generated bipartite graphs to study the correlation of layers and neuron types. A bipartite graph has two classes of nodes, for our purpose neuron types and neuropil layers. A neuron type projecting to a layer represents an edge. The resulting bipartite graph shows that neurons from the same community exhibit a wide repertoire of projection patterns that comprise several layers from different neuropils (Fig 7). This suggests that neurons from the same NB will be part of different neural circuits.

## Discussion

The upswing of Systems Biology has popularized and extended the use of graphs to analyze the structure of complex biological networks, from genomes to ecosystems. For instance, community detection techniques are useful for clustering highly interconnected nodes or predicting nodes function [37], [38]. In neural sciences, graphs have been particularly useful for the identification of hubs that interact with multiple nodes, acting as global communication centers [39, 40]. Analogous studies in *C*. *elegans* showed that “rich-club” neurons are connector hubs that play an integrative role in the communication between modules [28]. Community detection analysis also evidenced the modular structure of the *Drosophila* central brain [34, 35] as well as the “rich-club” concept [41, 42], seemingly conserved in different phyla.

Here we used the graph theory to set a method to analyze the lineage relationships of the neuron types of the optic lobes. We generated a large collection of clones to build graphs that represent the connections between the nodes of our collection. To strengthen this information we first considered biological relevant data within the clones, establishing a scoring system that resulted in the concept of reliability (R). R rates how likely a pair of neurons of a clone shares a common progenitor. We used the R-value to weigh the edges of our graphs and showed how it dramatically improves the quality of the community partitions and their predictive value. In our study, the occurrence alone does not produce good partition in communities due in part to the relatively small size of the sample. We overcome this limitation by using the inherent biological information provided by the reliability. We applied community detection tools to the R-weighed graphs to identify clusters of neurons with a tighter connection. Finally, we examined the modularity of the communities in our graphs to assess the goodness of the partitions, and analyzed with more detail our optimal modularity graph (R≥0,75). This graph shows 8 clusters of neurons that we posit correspond to the progenies of 8 distinct classes of neuroblasts in the optic lobes. Our results match previous lineage analysis of the communities C3, C4 and C5, and complete them with new neuron types. Most significantly, we find 5 new communities corresponding to 5 novel classes of NBs.

The confidence of our results is supported by the available data of the OL lineages. For instance, the neuroblasts of the tip of the larval outer proliferation center contribute neurons that innervate different layers of all three neuropils [17]. These neuron types include Mt8, Lccn2, and Lcn neurons that are all part of our community C5. Additionally, Lawf1 and Lawf2 appear in the community C3. These two neuron types are generated in the tips of a crescent with the same developmental origin. Both of them have the same glial siblings and share the expression of the transcription factors Homothorax and Eyes absent [30]. In this case we are revealing the lineage of a precursor in early larval stages (L2), before the tips develop, that later generates both Lawf neurons precursors. Finally, different T neurons (T2, T3 and T5), which are the central core of the community C4, are part of the same lineage [36]. These results indicate the high confidence of our analysis.

Some of our clones show sparse cell bodies distribution, consistent with the cell migration of Lawf1 and Lawf2 neurons [30] and neural progenitors [36]. This active cell migration, a common feature during the development of the mammalian brains [43, 44] accounts for an extensive reorganization during circuit formation in the *Drosophila* OL [18] as opposed to the central brain, where cell bodies generated by the same NB remain in spatially restricted compartments [34, 35].

One possible weakness of our analysis is the presence of Tm neurons in the C4 community that also includes T2, T3 and T5 neuron types. Tm neurons are likely to arise from neuroblasts from the outer proliferation center [18], whereas T-C cells are part of neuroblasts from the inner proliferation center [36]. This discordance may be the result of the relatively small size of our clone sample, leading to few nodes assigned to a dubious community. Generating clones is extremely labor consuming due to the intrinsic constraints of using pan-neuronal drivers. While our community analysis shows 65 cell types for an R of 0.25, the number of cell types decreases to 42 for the optimal R (≥0.75). Despite these limitations, rather than a developmental analysis, we aim to set a method that can be applied to other complex systems where the acquisition of data is tedious or technically limited, and it is possible to define an R. In these cases, the application of our method will help to establish accurate approximations. In addition, our protocol allows for incorporation of new data leading to a comprehensive catalogue of the lineage relationships of the neuron types of the optic lobe of *Drosophila*. Future data from existing or newer lineage tracing techniques [3, 45] will contribute towards this end.

Understanding the structure of the fly brain circuits may help explaining how it functions. In the human brain, where structure-function correlation has been widely studied, areas or nodes with similar patterns of connections tend to share similar functions [29, 46]. Analogously, in the *Drosophila* central brain lineage related modules form brain structures that are involved in the same sensory pathway [34, 35]. However, although some graphs show that neurons within a community are part of the same functional circuits (e.g. T5 and T2 neurons), our bipartite graphs indicate no correlation between the neurons of a community and their functionality. This establishes a difference between optic lobes *versus* central brain regions in the *Drosophila* brain, as well as a link with mammal brain development. Similar to the fly optic lobes, in the mice brain most of the forebrain interneurons share a common progenitor and then migrate to sparse brain structures contributing to different circuits [47, 48]. The developmental similarities of vertebrates and invertebrates and the existing lineage tracing techniques in mice permit the application of this method in their higher nervous systems, where bipartite graphs will be useful for large-scale analysis of circuit formation. Defining the variables that result in the R to each particular case will help deducing the cell lineage of the hundreds of neuron types of these complex nervous systems. The methodology that we develop here will also allow identifying related clusters of nodes in other systems with restricted sampling and where an R can be defined.

## Material and methods

### Descriptive parameters of the clones

We annotated in the S1 Table the following parameters for each clone. *Size*: indicates the number of cells present in the clone. We establish a binary system for the next three parameters. *Symmetry*: “1” indicates clones with two equal populations of neurons (in size and cell type) and “0” indicates non-symmetric clones. *Homogeneity*: “1” indicates that all the cells are of the same cell type (homogeneous clone) versus “0” (heterogeneous clone), indicating several cell types in the clone. *Quality (q)*: the expression intensity of GFP or RFP in fine neuronal processes varies in different clones. We ranked our clones from “0”, for clones where the stainings were not uniform and the identification of neurons may lead to confusion due to the similarity between certain neuron types. “2” indicates that the neurons are clearly identifiable. “3” and “4” indicate clones of exceptional quality, with a bright fluorescent signal. *Unidentified neurons*: “1” indicates that some neurons of the clone remained unidentified, whereas “0” indicates all neurons can be recognized. Clones with unidentified neurons can be easily discarded for high threshold analysis, however our analysis in pairs allows us to use them, as this fact does not affect the results.

#### Clones scoring method

For all the clones analyzed in our study (S1 Table) we score each of them as follows.

#### Clones with one cell type

Homogeneous cell clones, either sister cells (Fig 3A and 3B) or larger (Figs 1B–1D and 3C) get the highest score (R = 1) because they are arguably the progeny of a dedicated NB. The R for these clones only depends on the q (S2 Table).

#### Clones with two different cell types

1. For clones with two different cell types of the same color the strength of the relationship decreases with the number of neurons (S3 Table). We assume that larger clones are the product of more than one NB. We rate with maximum score (0.95) clones of few cells (1–4 cells of each cell type). The score is slightly reduced as the number of neurons increases, but the strength remains high for up to 20 neurons. We reason that when one GFP or RFP is lost in the clone (S1B Fig), the result is a clone with neurons labeled with one color that are very likely related regardless of the number of cells or cell types. However, we penalize this relationship as the number of cells increase, so clones with more than 20 cells get lower score (0.50). Although in some cases we might be penalizing cell types potentially related, we increase the robustness of the analysis using a conservative approach. Besides, if these cell types are recurrent in other clones, their relationship will be restored. 2. For clones with two different cell types and two colors (S4 Table) the score decreases more significantly because each color-related population might be the product of a NB. Although this is very unlikely for sister cell clones (which we score 0.95) (Fig 3A), this likelihood increases with the number of neurons. 3. If one of the neuron types is single-colored and the other is bicolored we apply S3 Table to the single-colored type, as established previously, and we apply a correction factor proportional to the number of bicolored neurons (S5 Table). This correction is inversely proportional to the number of neurons. 4. In a few cases we see clones with two cell types, each of them labeled by both colors. If there is symmetry (Fig 1D and 1E and S1C Fig) between the cell types (i.e. (a, a), (b,b), Fig 3D), we give a high score (0.8), as this is a strong indicator of lineage relationship. If there is no symmetry, we apply a strong correction factor (S6 Table) that penalizes proportionally to the total number of neurons. We reason that even for relatively small cell clones of this kind, the color code indicates that each cell type correspond to an independent NB.

#### Clones with more than two cell types

Clones in the optic lobe, with a population of around 66,000 neurons of more than 80 cell types, can display an overwhelming number of combinations. The chances of getting duplicates of samples with more than two cell types are extremely low, requiring hundreds of thousands of samples for a complete analysis. For this reason, as most of our clones have more than two cell types (Fig 3E), we propose a study in pairs. Each pair of neuron types is subjected to the scoring detailed in the previous section and we add a correction factor proportional to the number of neuron types and the total number of neurons (S7 Table). We set the maximum score for two neuron types and penalize the relationship as the number of cell types increase. The penalization is stronger when the total number of neurons in the clone is larger than 20. We set this benchmark reasoning that the probability of labeling the last 5 rounds of division of two independent NBs (generating 10 neurons each) is extremely low. In other words, larger clones have more chances of being the product of more than one NB.

For a flow chart summarizing the calculation of the R for each pair of neuron within a clone see S10 Fig. We also performed a statistical analysis of the R by pairs of neuron types (S1 Appendix).

### Calculation of the total reliability

For the summation of independent events we follow the inclusion-exclusion principle:

$$r_{ij}=\sum_{\emptyset \neq K\subseteq \left\{1,2,\ldots ,n\right\}}{\left(-1\right)}^{\left|K\right|-1}\prod_{k\in K}r_{ij}^{k}$$ (1)

For instance, if i and j are present in the clones “1”, “2” and “3”, we use the equation:

$$r_{ij}=r_{ij}^{1}+r_{ij}^{2}+r_{ij}^{3}-r_{ij}^{1}r_{ij}^{2}-r_{ij}^{1}r_{ij}^{3}-r_{ij}^{2}r_{ij}^{3}+r_{ij}^{1}r_{ij}^{2}r_{ij}^{3}$$ (2)

### Community detection algorithm

To know whether our graphs have a community structure we use the widely tested null-model [49], where the expected degree sequence equals that of our graph. Based on this model, we can quantify the quality of the community structure through the modularity function.

We use the null model to compute the probability of an edge linking two vertices with the equation:

$$Q=\frac{1}{2\left|E\left(G\right)\right|}\underset{ij\in V\left(G\right)}{\sum }\left(A_{ij}-\frac{d_{i}d_{j}}{2\left|E\left(G\right)\right|}\right)\delta \left(C_{i},C_{j}\right)$$ (3)

where *i*,*j* go over all the vertices of the graph G = (V,E), d_i_ represents the degree of the vertex *i* and C_i_ the community to which *i* belongs. The summatory only considers edges from the same community, so we can use the equation:

$$Q=\overset{n_{c}}{\underset{c=1}{\sum }}\left[\frac{l_{c}}{\left|E\left(G\right)\right|}-{\left(\frac{d_{c}}{2\left|E\left(G\right)\right|}\right)}^{2}\right]$$ (4)

Where *n*_*c*_ is the total number of communities and *I*_*c*_ and *d*_*c*_ are, respectively, the number of edges and the degrees summatory of the vertices within the community *c*. Therefore, if the communities are formed by complete graphs, the modularity is 1, whereas modularity in random graphs is 0.

All the graphs of our study were built using the program Mathematica. The calculation of the communities was done with the library iGraph on the programming languages Python and C. The algorithm solves large integer optimization problems and finds the optimal modularity score as well as the corresponding community structure. The calculation time ranged from the hundredth of seconds for simple graphs, to 50 hours for the most complex ones. We used a Mac Pro 6.1 equipped with a Quad-Core Intel Xeon E5, OS Mac OSX 10.9.5, 3,7 GHz processer and 4 nucleus.

### Fly strains

For twin spot MARCM, (*elav*) *c155-gal4; FRT40A*,*UAS-CD8*::*GFP*,*UAS-rCD2-miRNA/CyO*,*y+*, *yw; FRT40A*,*UAS-CD8*::*GFP*,*UAS-rCD2-miRNA/CyO*,*y+* or *actin5c-gal4/TM6b* males were crossed with *hsFLP; FRT40A*,*UAS-rCD2RFP*,*UAS-GFP-miRNA/CyO*,*y+* females. MARCM clones were generated by crossing *hsFLP*, *UAS-CD8*::*GFP; FRT42D*, *tub-Gal80; tub-Gal4/TM6B* females with *y*,*w; FRT42D/CyO; TM2/TM6B* males (stocks were gifts from T. Lee).

### Brain immunostainings

Twin spot MARCM and MARCM clones were induced between 48 and 72 hours larval stage (early L2 to early L3), by heat shocking the vials at 37°C during 8 to 20 minutes. No significant difference was detected in the clones generated in this interval. The brains were dissected in 1xPBS from 3–7 days old adult flies. After dissection they were placed on glass wells on ice and fixed in PBS and 4% paraformaldehyde during 1h in the orbital shaker. Fixed brains were incubated in the primary antibody solution (PBS, 01% triton X-100, GFP, RFP and N-Cadherin; for MARCM clones RFP is replaced by chaoptin antibody) overnight at RT. The solution was washed with PBST three times and secondary antibody solution (PBS, 01% triton X-100, Alexa 555 anti-rabbit or anti-mouse, Alexa 488 anti-rabbit or sheep, Alexa 647 anti-rat or antimouse) was added during 5 hours. Brains were washed 3 times in PBST and mounted on the slides with vectashield (Vector Laboratories, H-1000). Primary antibodies: rabbit anti GFP 1:1000 (molecular probes A11122), sheep anti GFP 1:1000 (AbD Serotec), mouse anti RFP 1:500 (MBL International), rabbit anti-DS-red 1:1000 (Clontech). Chaoptin anti mouse and N-cadherin anti rat (both 1:25; Hybridoma bank). Secondary antibodies: Alexa 488 donkey anti-rabbit (1:1000), Alexa 488 donkey anti-sheep (1:1000), Alexa 555 donkey anti-mouse (1:500), Alexa 555 donkey anti-rabbit (1:500), Alexa 647 donkey anti-rat (1:200) and Alexa 647 donkey anti-mouse (1:200), all secondary antibodies from Molecular Probes.

### Microscopy imaging

Images of the clones were acquired using a Leica SP5 confocal laser scanning microscope and stack projections processed with Leica AF-Lite and Fiji softwares.

## Supporting information

S1 Fig Models of NB division and resulting clones using the twin spot MARCM technique. (A) The left panel shows in red and green the resulting progeny of a GMC clone. The central and right panels display the color distribution of the resulting progeny from a NB dividing asymmetrically. (B) In the events of cell death, clones can display only one color. (C) Model showing the resulting lineage of a progenitor (neuroblast or neuroepithelial cell) dividing symmetrically.
(PDF)

S2 Fig Calculation of the R in a sample clone. We show the different pairs of neurons within the clone in Fig 3E and calculate the R for each of them according to the S2–S7 Tables. t2-t7 indicate the (S2 to S7) Table applied to each pair. The value 0.72, obtained from S2 Table and the total number of neurons in a clone (S6 Table), multiplies every pair value to obtain the final R for each pair.
(PDF)

S3 Fig Circular representation of the occurrence graph of the clones. This graph is a circular version of the graph in Fig 2.
(PDF)

S4 Fig Weighed graphs. Random (A) and circular (B) weighed graphs of the entire clone collection using the R instead of the occurrence as a weigh for the edges. The thickness of the edge between two nodes is proportional to the R.
(PDF)

S5 Fig Discretized weighed graphs. Weighed graphs for values of R≥0 (A) and R≥0.95 (B).
(PDF)

S6 Fig Community graphs for discretized R values. Graphs built using community detection algorithm for R values ≥ 0 (A), 0.25 (B), 0.5 (C), 0.9 (D) and 0.95 (E).
(PDF)

S7 Fig Adjacency matrix for R = 0. (A) Adjacency with all the clones of our collection distributed randomly or ordered by communities (B).
(PDF)

S8 Fig Discretized occurrence community graphs. (A) Graph showing community structure of neuron types that appear together at least one time (occurrence = 1). (A) Graph for occurrence = 2. (B). Graph for occurrence = 3 (C). Graph for occurrence = 4 (D).
(PDF)

S9 Fig Neuronal projections in the optic lobe. Axonal projections of the neuron types from the 8 communities to the different layers of the optic lobe (layers 1, 1–10, 1–6, 1–4 of the lamina, medulla, lobula and lobula plate neuropils).
(PDF)

S10 Fig Flow chart for the calculation of the R for each pair of neurons. The flow chart shows all the possible combinations of pairs of neurons in our clones and the correction coefficients to be applied from S2–S7 Tables.
(PDF)

S1 Appendix Statistical analysis of R. Distribution of R for all pairs of cells, number of cell types in each clone, distribution versus number of clones, mean, standard deviation and quartiles are shown.
(PDF)

S1 Table Clone collection. This table contains all the clones analyzed in our study. Each file corresponds to one clone and the color of the number indicates the cells of a specific type in the clone. To compute large clones we use the value 100, if there are between 60 and 100 cells of one type and 200 for larger values.
(PDF)

S2 Table Clones with one cell type. (PDF)

S3 Table Clones with two cell types of the same color. (PDF)

S4 Table Clones with two cell types of different color. (PDF)

S5 Table Clones with two neuron types of different color. (PDF)

S6 Table Clones with two neuron types of two colors. (PDF)

S7 Table Clones with more than two neuron types. (PDF)
