# Supplementary figures and images for: A network approach to analyze neuronal lineage and layer innervation in the Drosophila optic lobes
Source: PLoS One. 2020 Feb 5;15(2):e0227897. doi: 10.1371/journal.pone.0227897 (PMC7001925; doi:10.1371/journal.pone.0227897)

# Twin-Spot MARCM

NB asymmetrical division

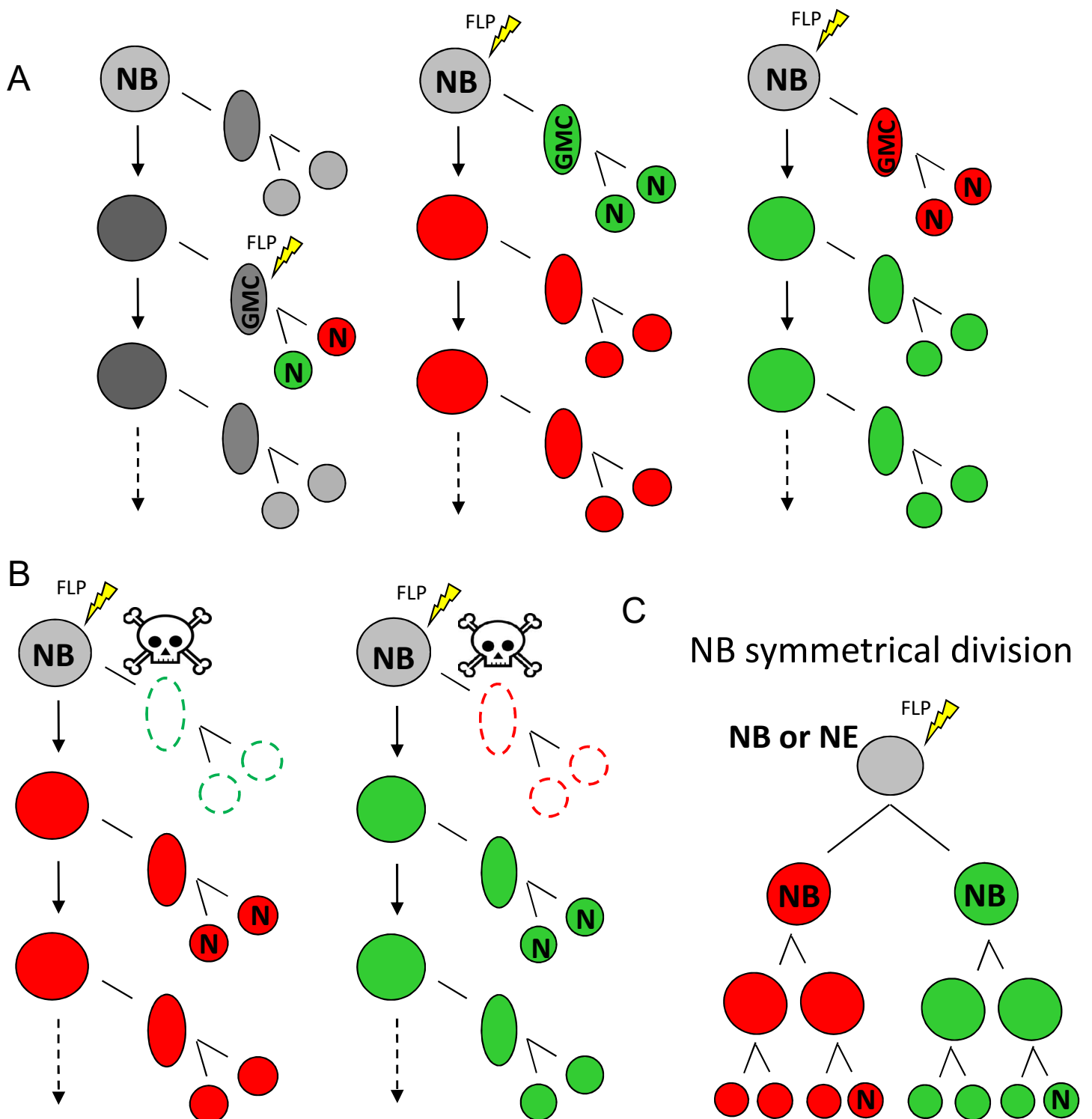

S1 Figure

Supplement: S1 Fig — (A) The left panel shows in red and green the resulting progeny of a GMC clone. The central and right panels display the color distribution of the resulting progeny from a NB dividing asymmetrically. (B) In the events of cell death, clones can display only one color. (C) Model showing the resulting lineage of a progenitor (neuroblast or neuroepithelial cell) dividing symmetrically. (PDF) [file pone.0227897.s001.pdf]

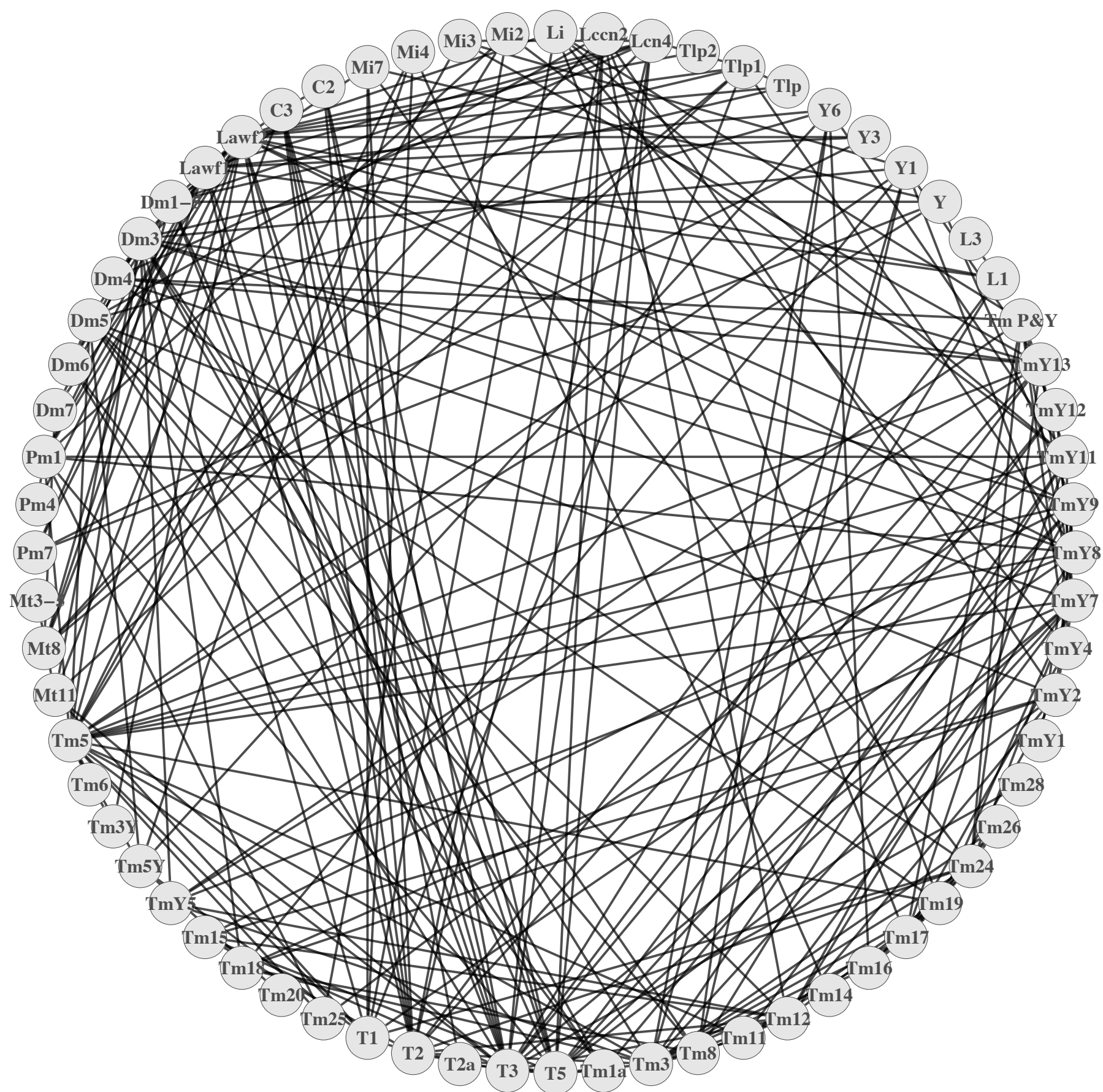

**S3 Fig**

Supplement: S3 Fig — This graph is a circular version of the graph in Fig 2. (PDF) [file pone.0227897.s003.pdf]

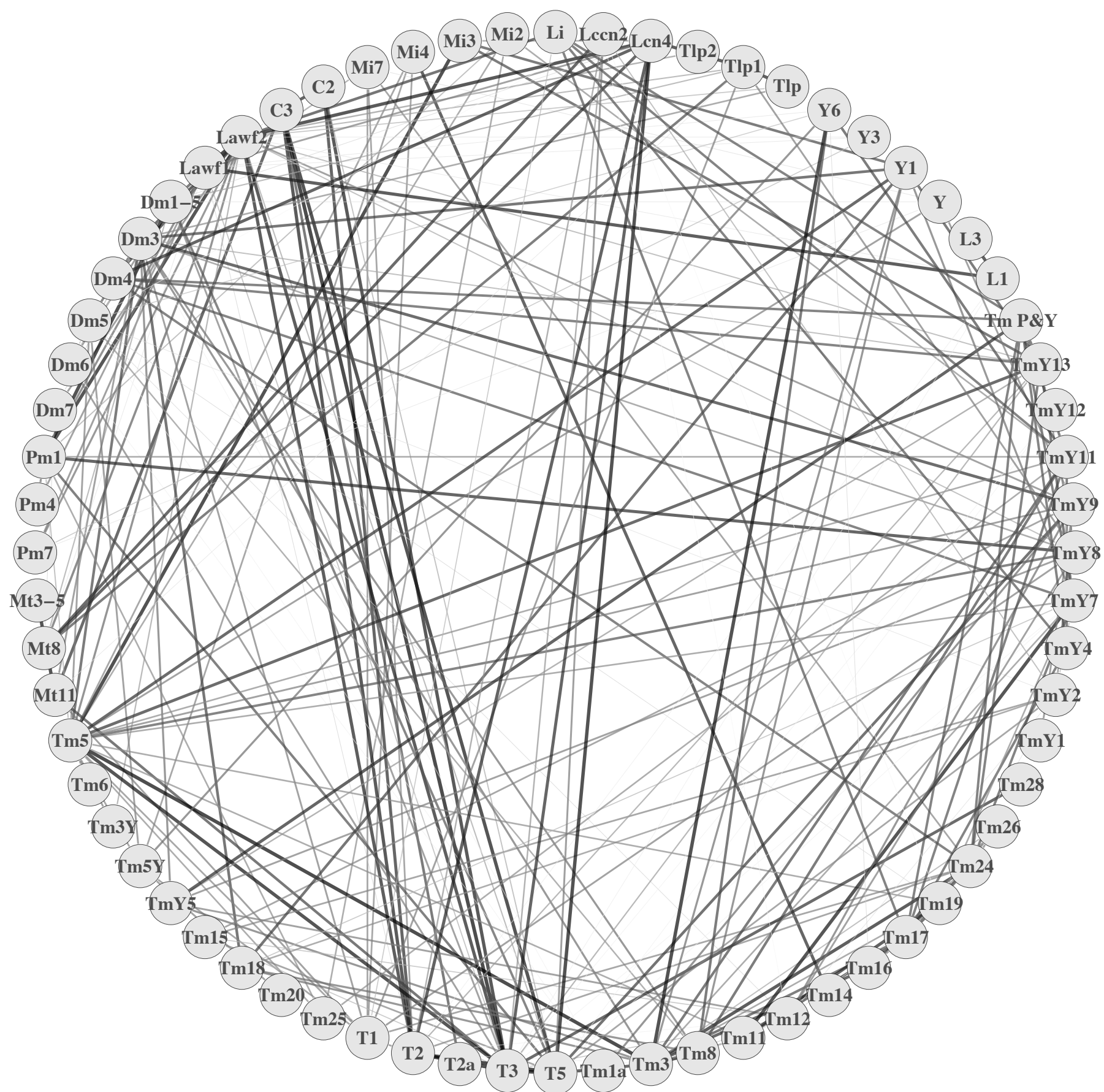

**S4B Fig**

Supplement: S4 Fig — Random (A) and circular (B) weighed graphs of the entire clone collection using the R instead of the occurrence as a weigh for the edges. The thickness of the edge between two nodes is proportional to the R. (PDF) [file pone.0227897.s004.pdf]

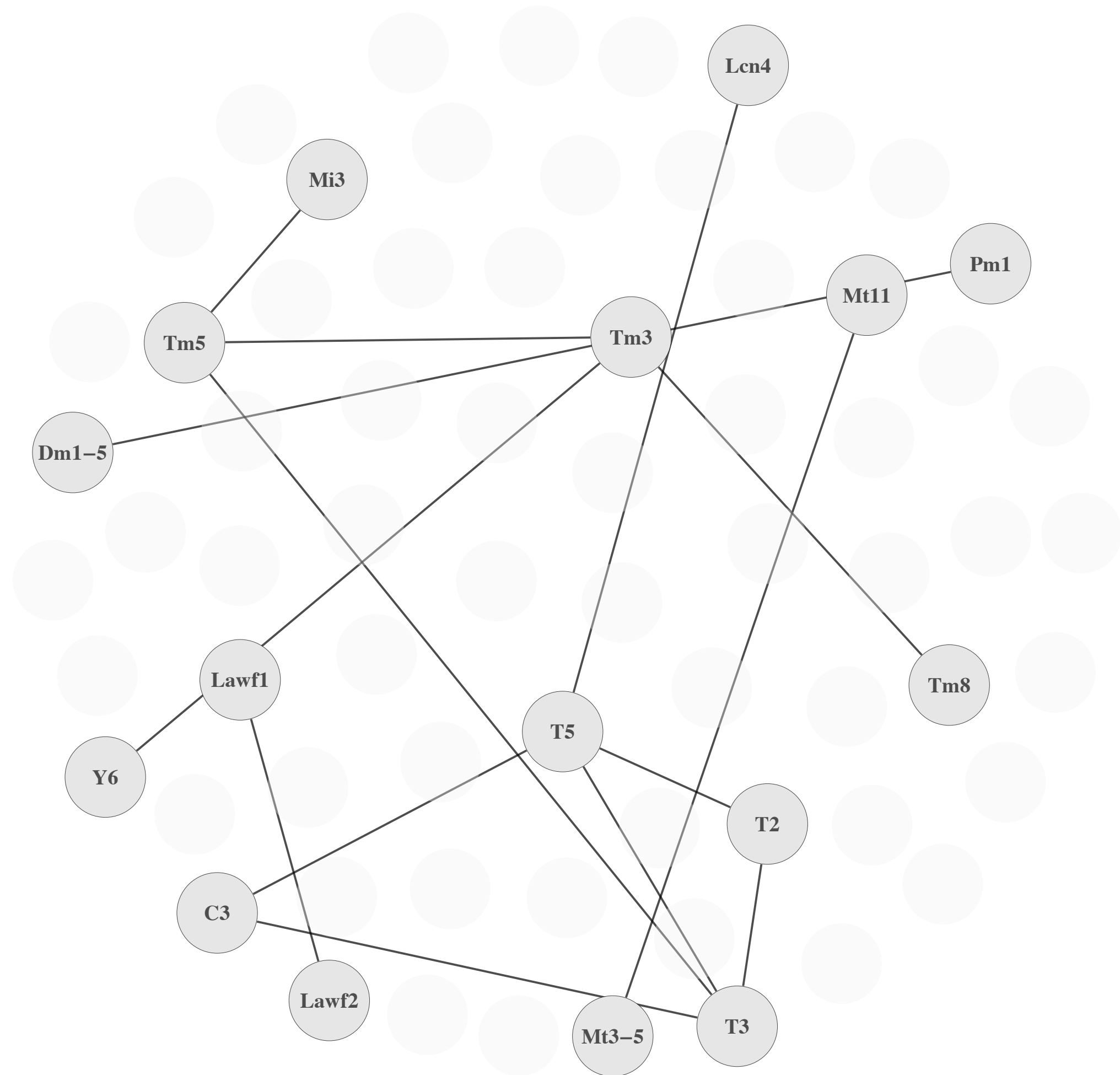

**S5B Fig**

Supplement: S5 Fig — Weighed graphs for values of R≥0 (A) and R≥0.95 (B). (PDF) [file pone.0227897.s005.pdf]

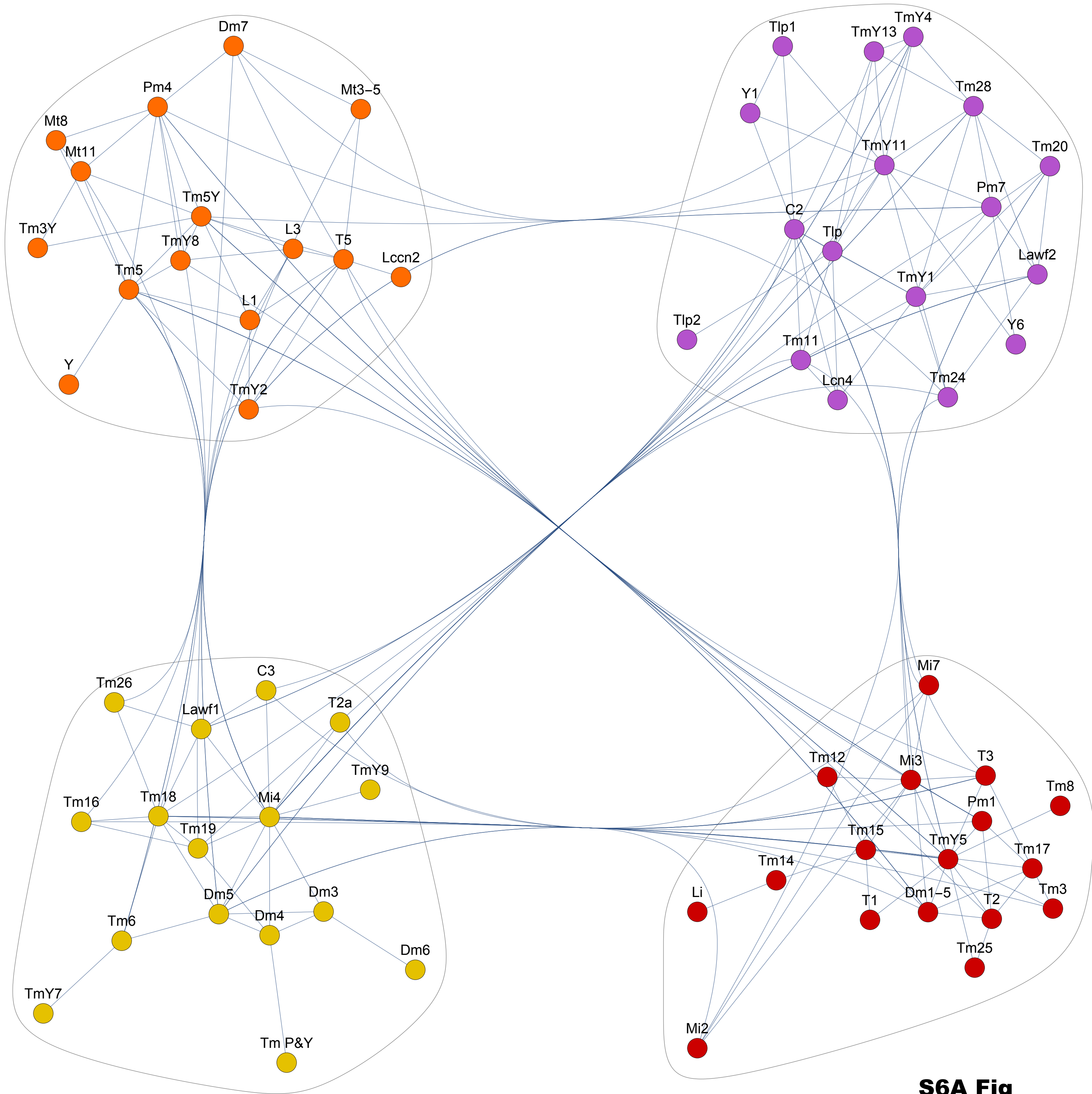

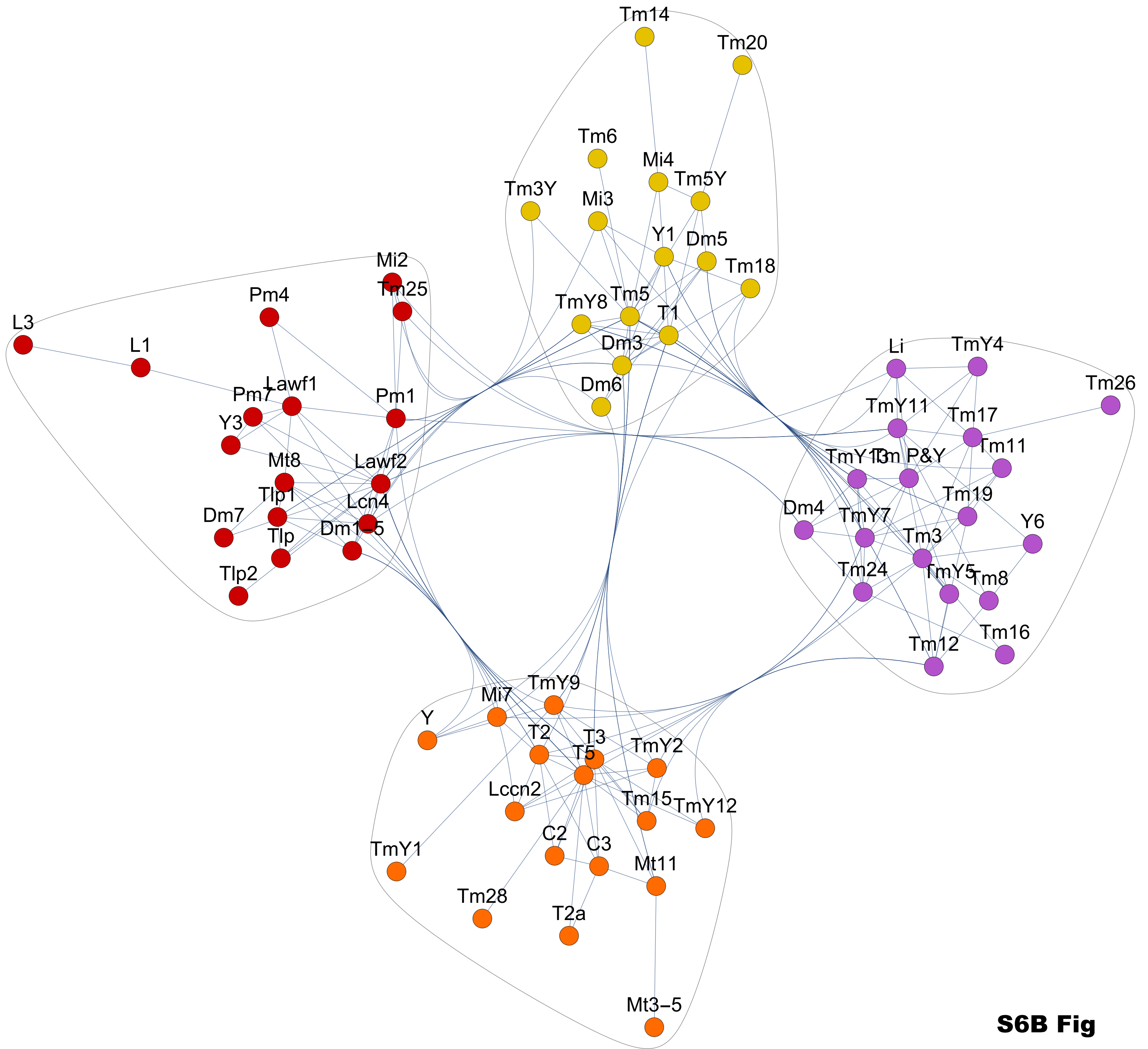

**S6B Fig**

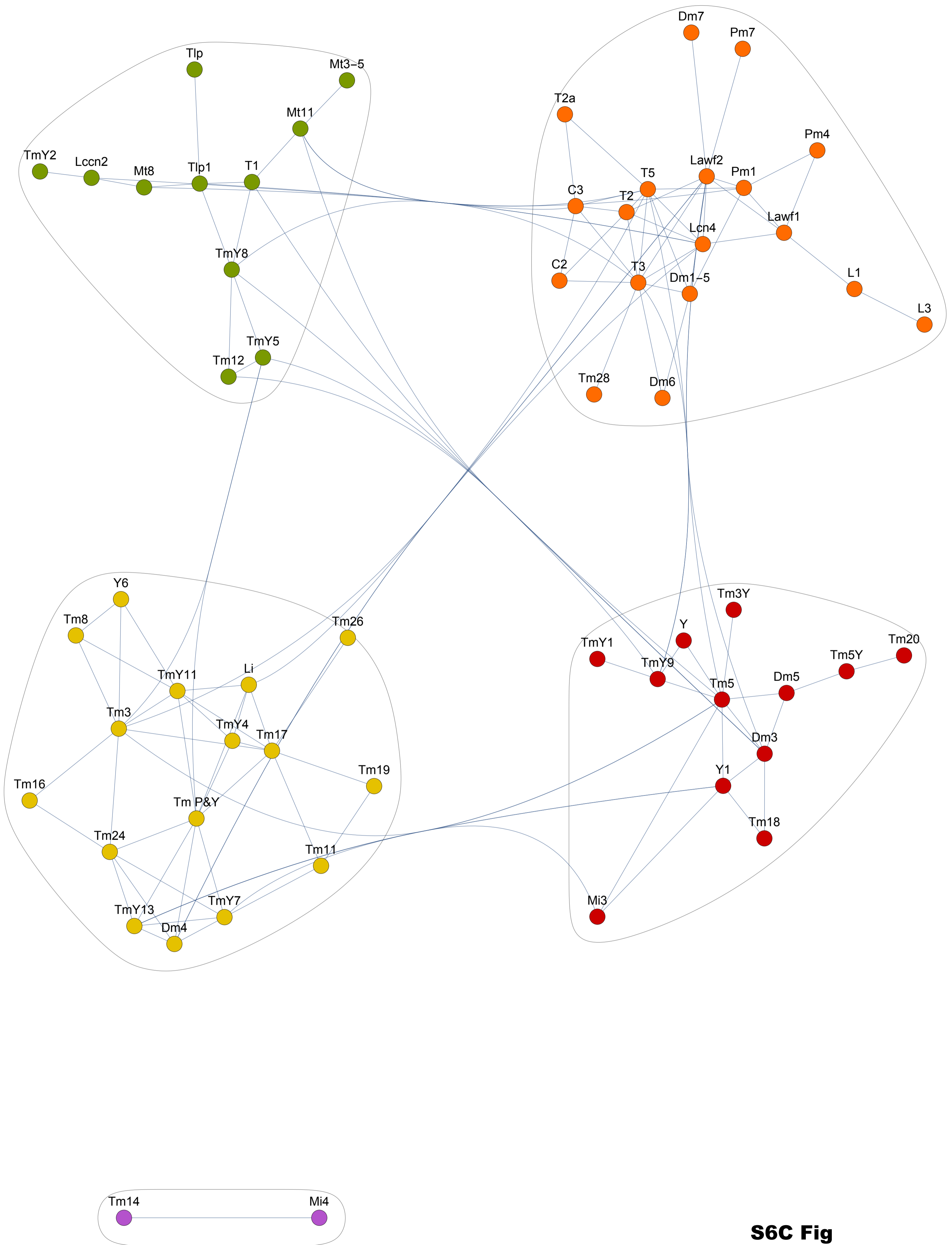

**S6C Fig**

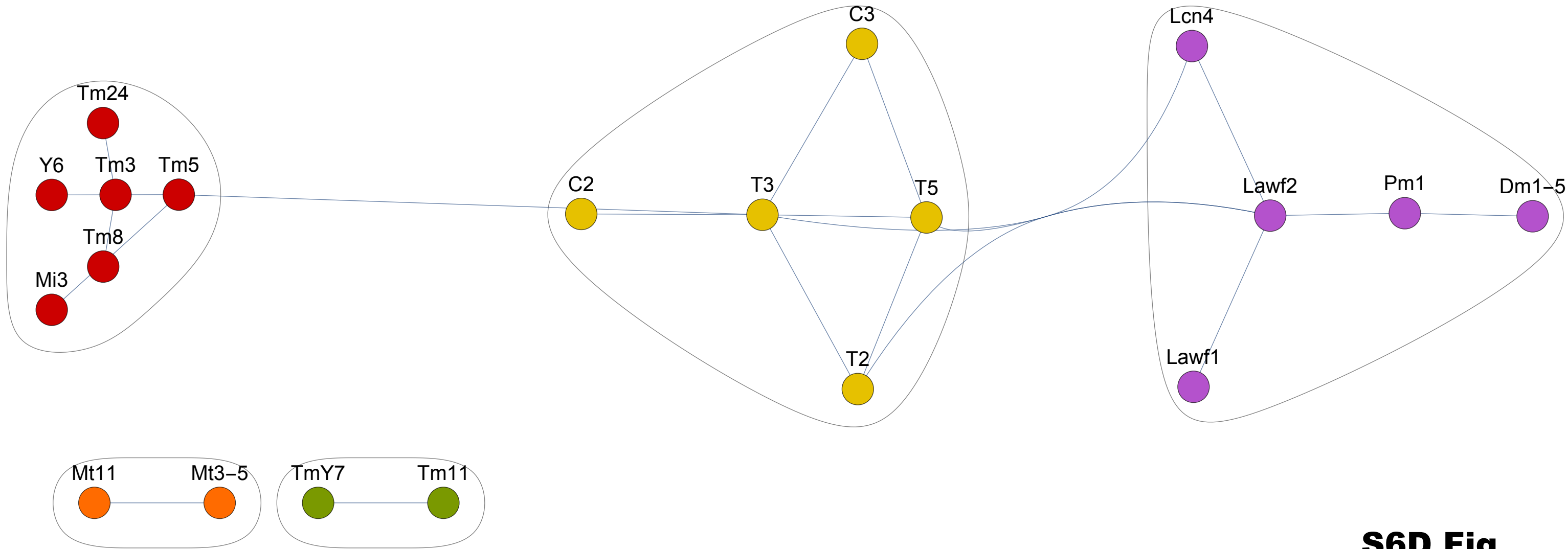

**S6D Fig**

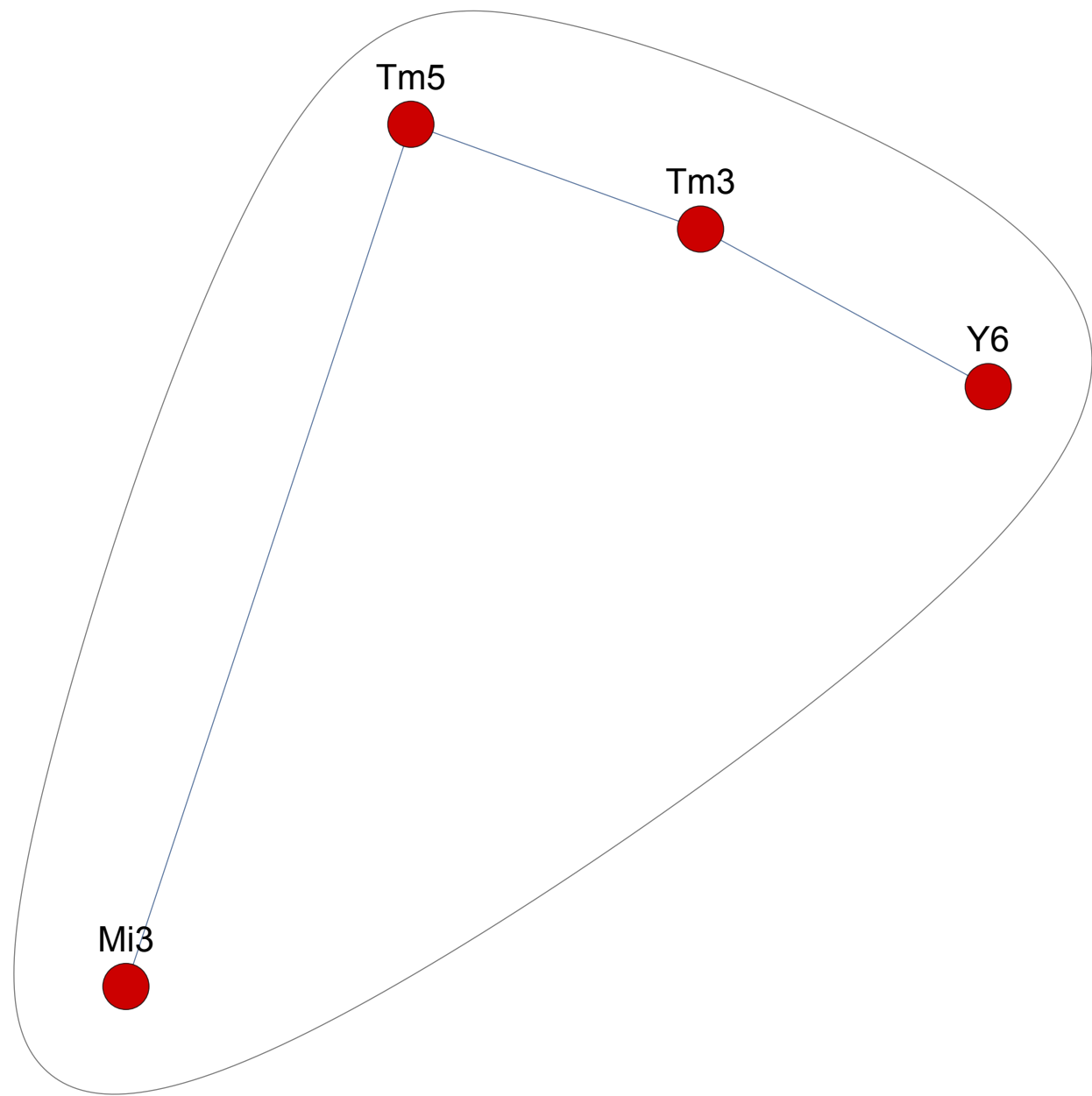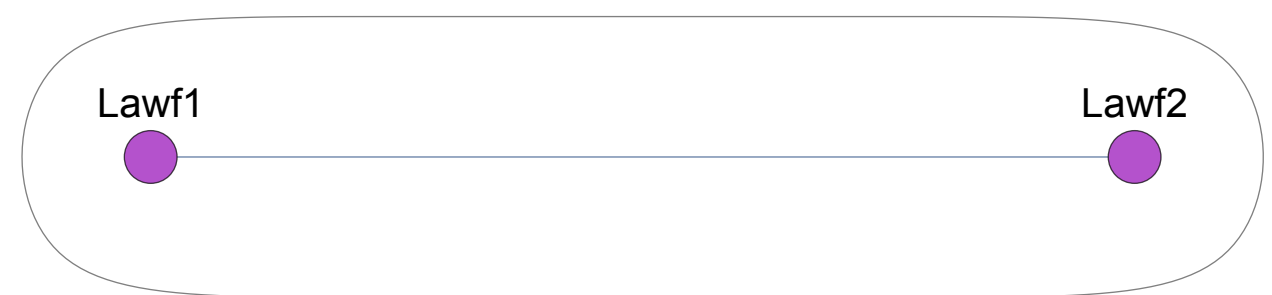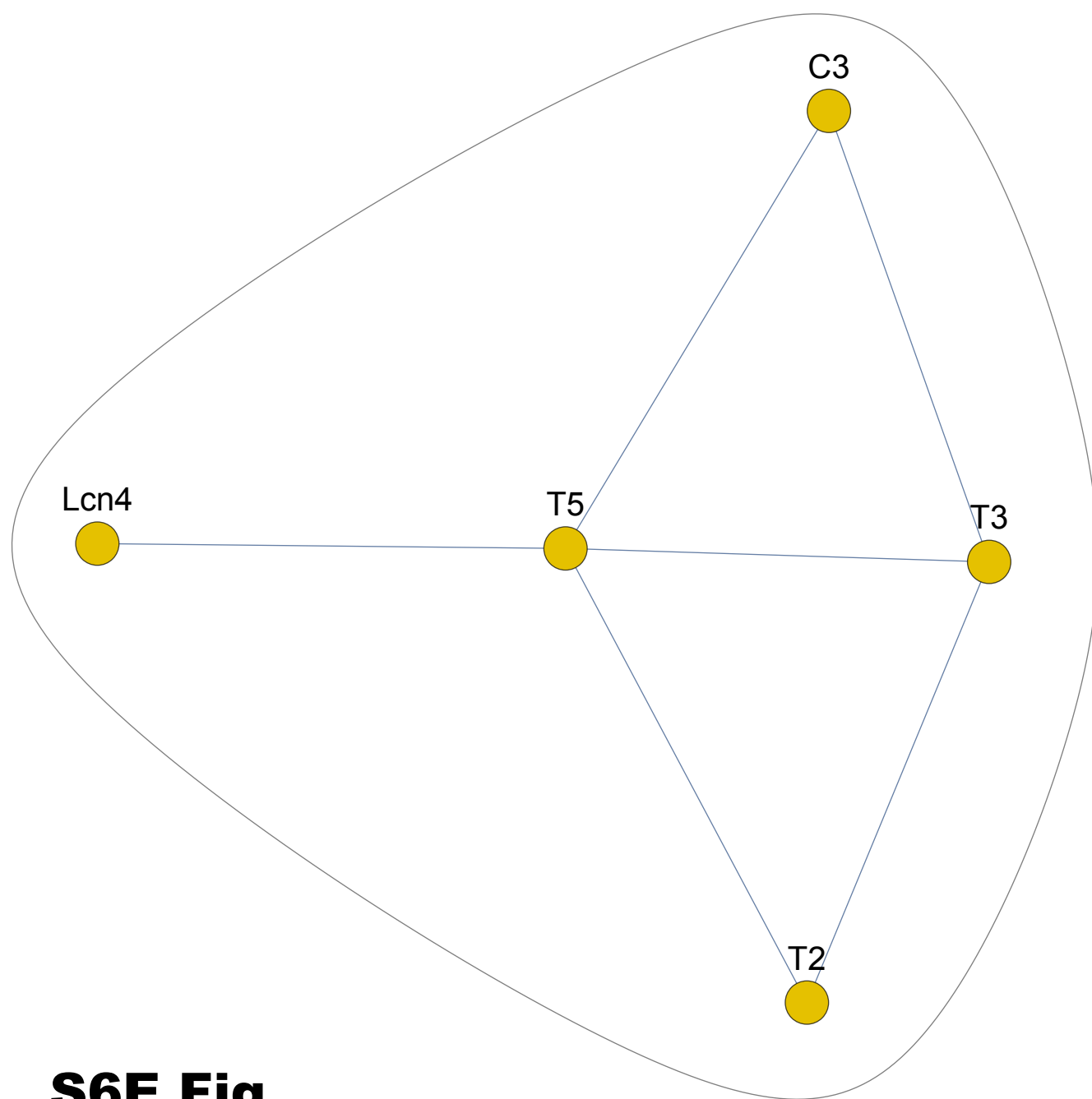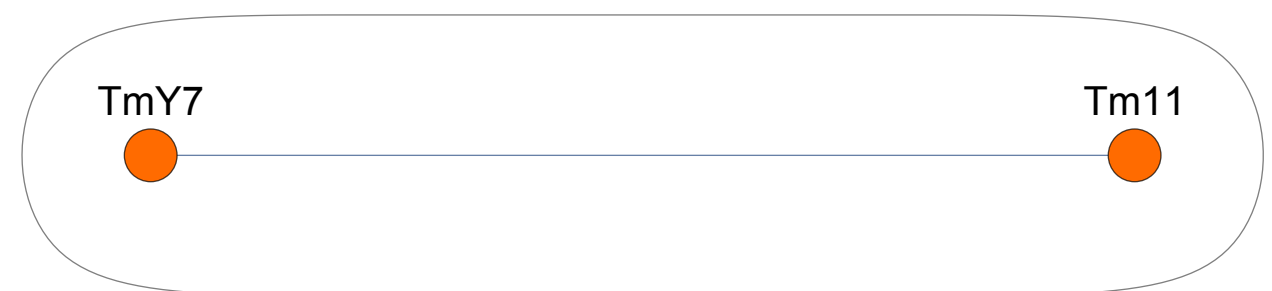

**S6E Fig**

Supplement: S6 Fig — Graphs built using community detection algorithm for R values ≥ 0 (A), 0.25 (B), 0.5 (C), 0.9 (D) and 0.95 (E). (PDF) [file pone.0227897.s006.pdf]

A

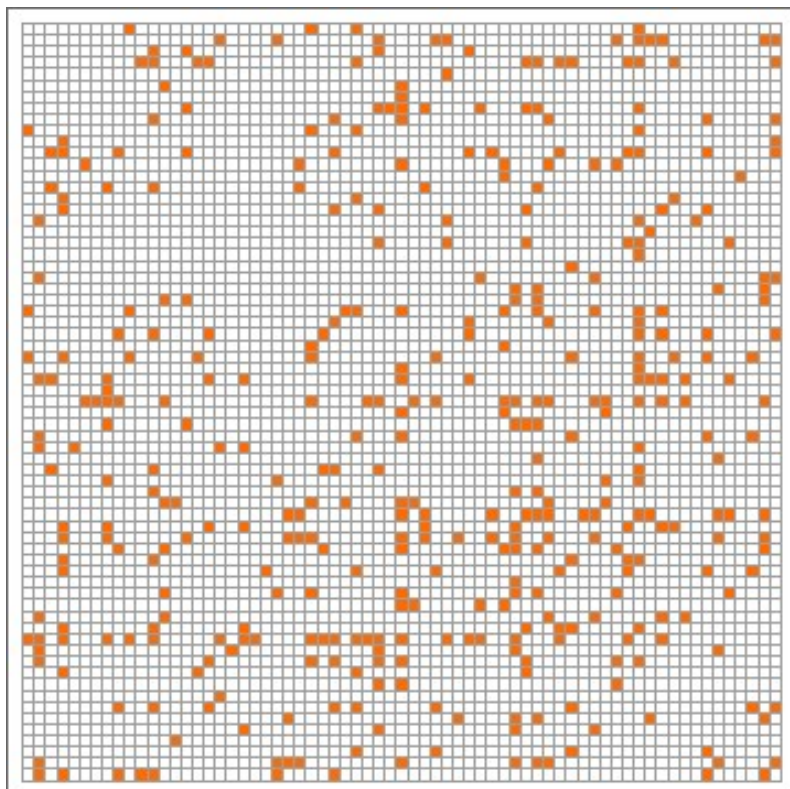

B

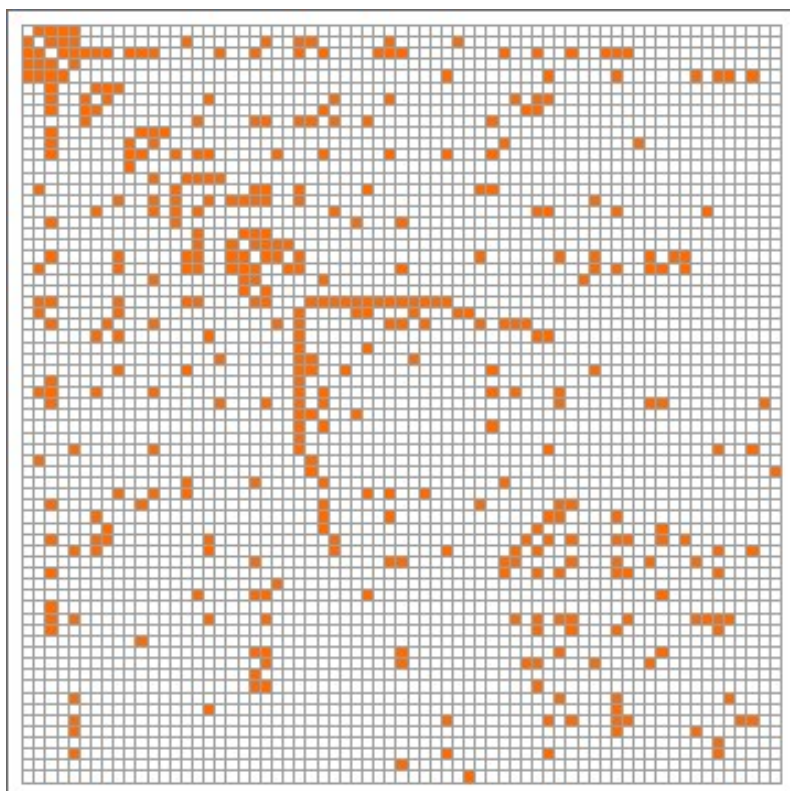

$r=0$

Supplement: S7 Fig — (A) Adjacency with all the clones of our collection distributed randomly or ordered by communities (B). (PDF) [file pone.0227897.s007.pdf]

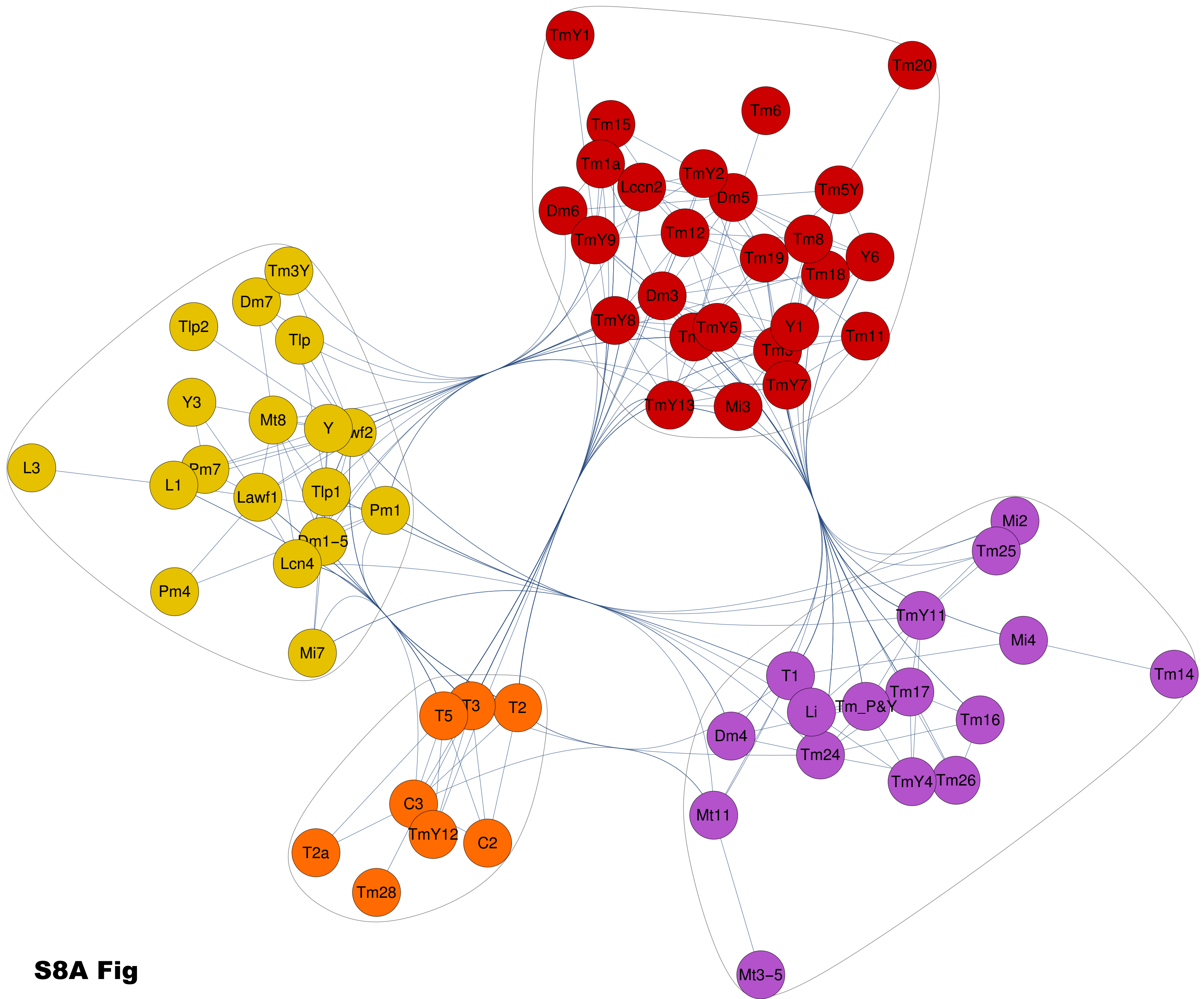

**S8A Fig**

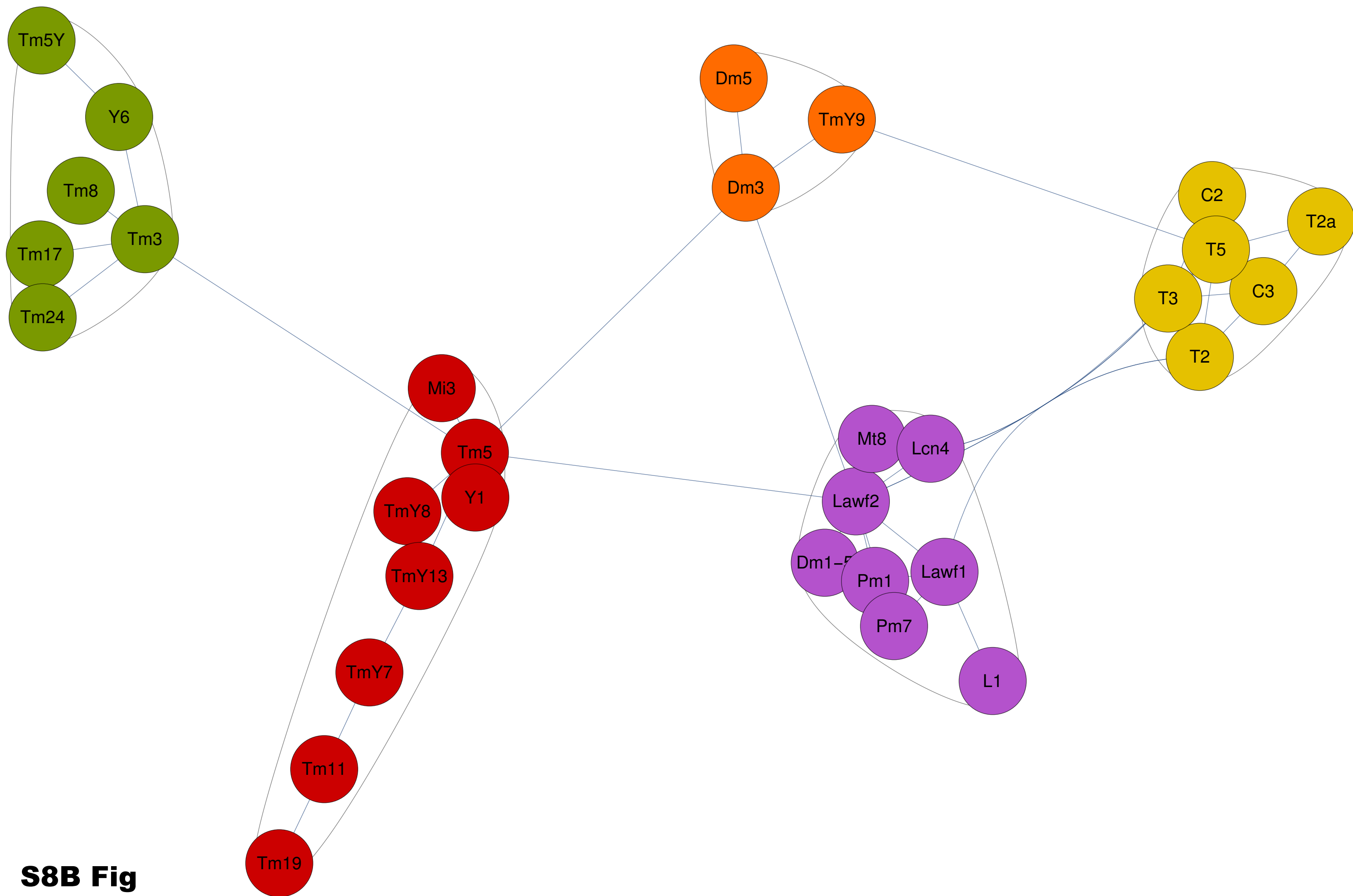

**S8B Fig**

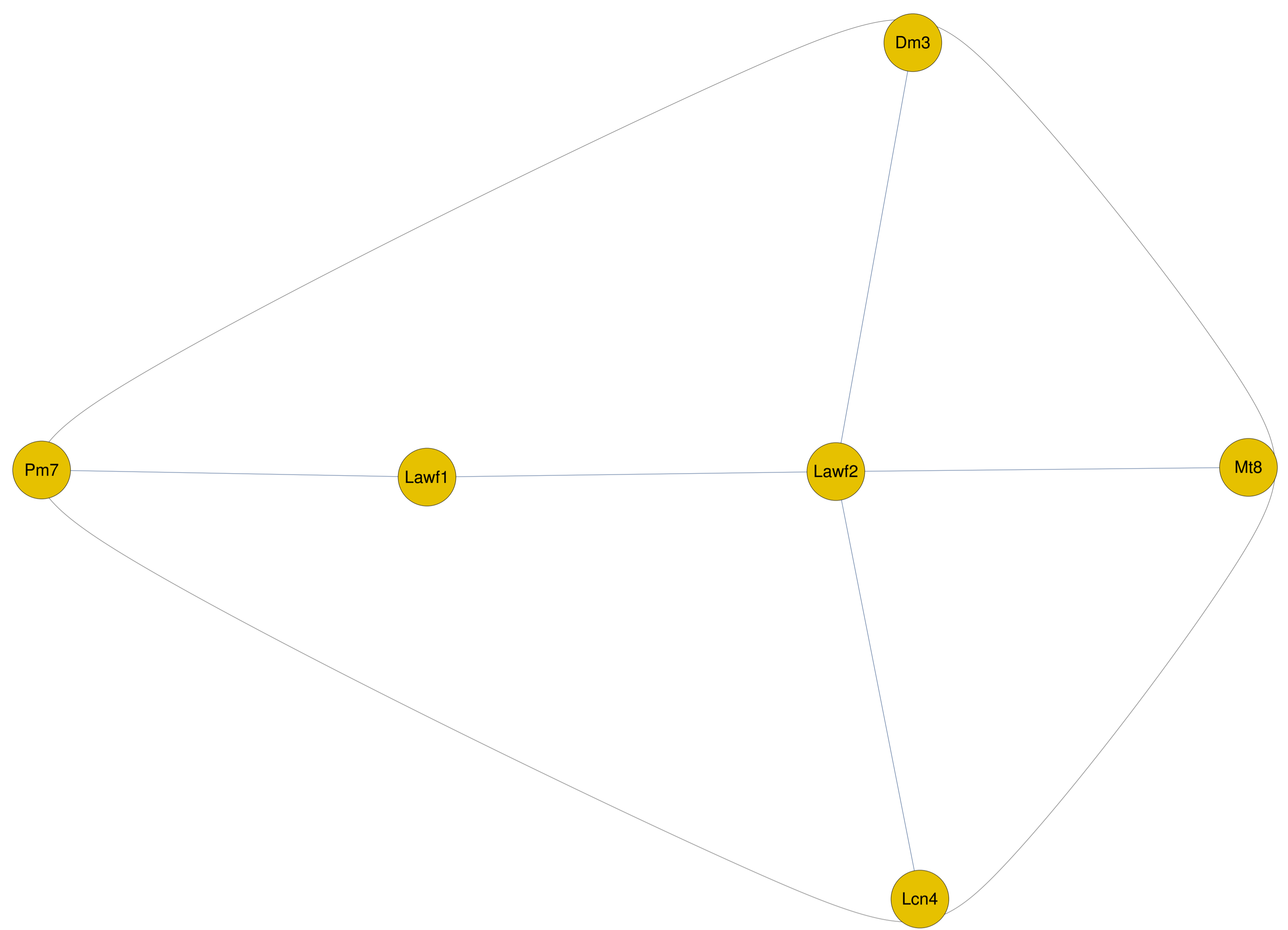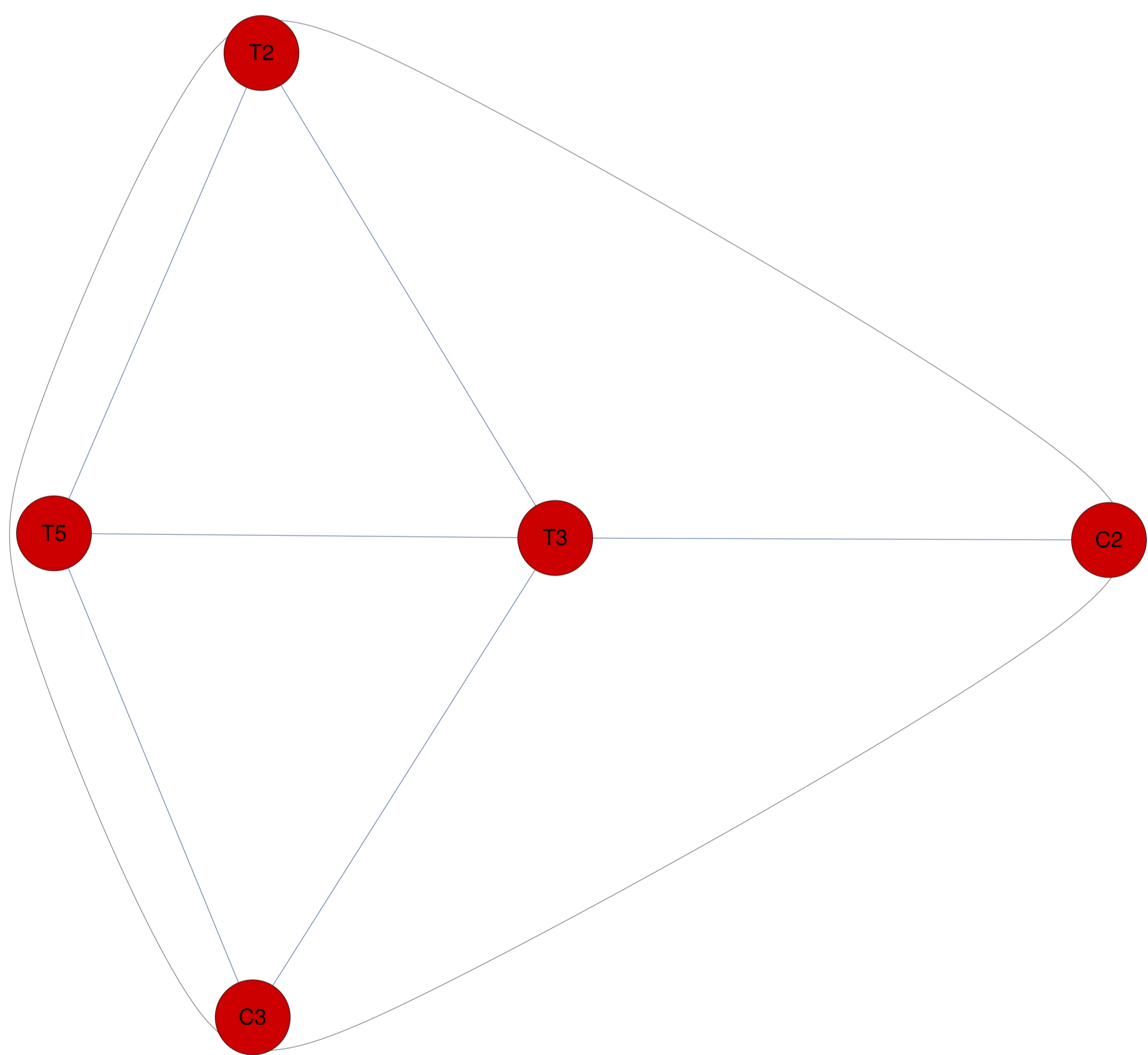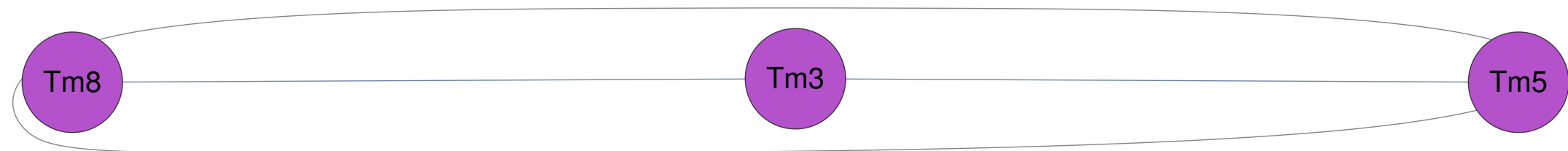

**S8C Fig**

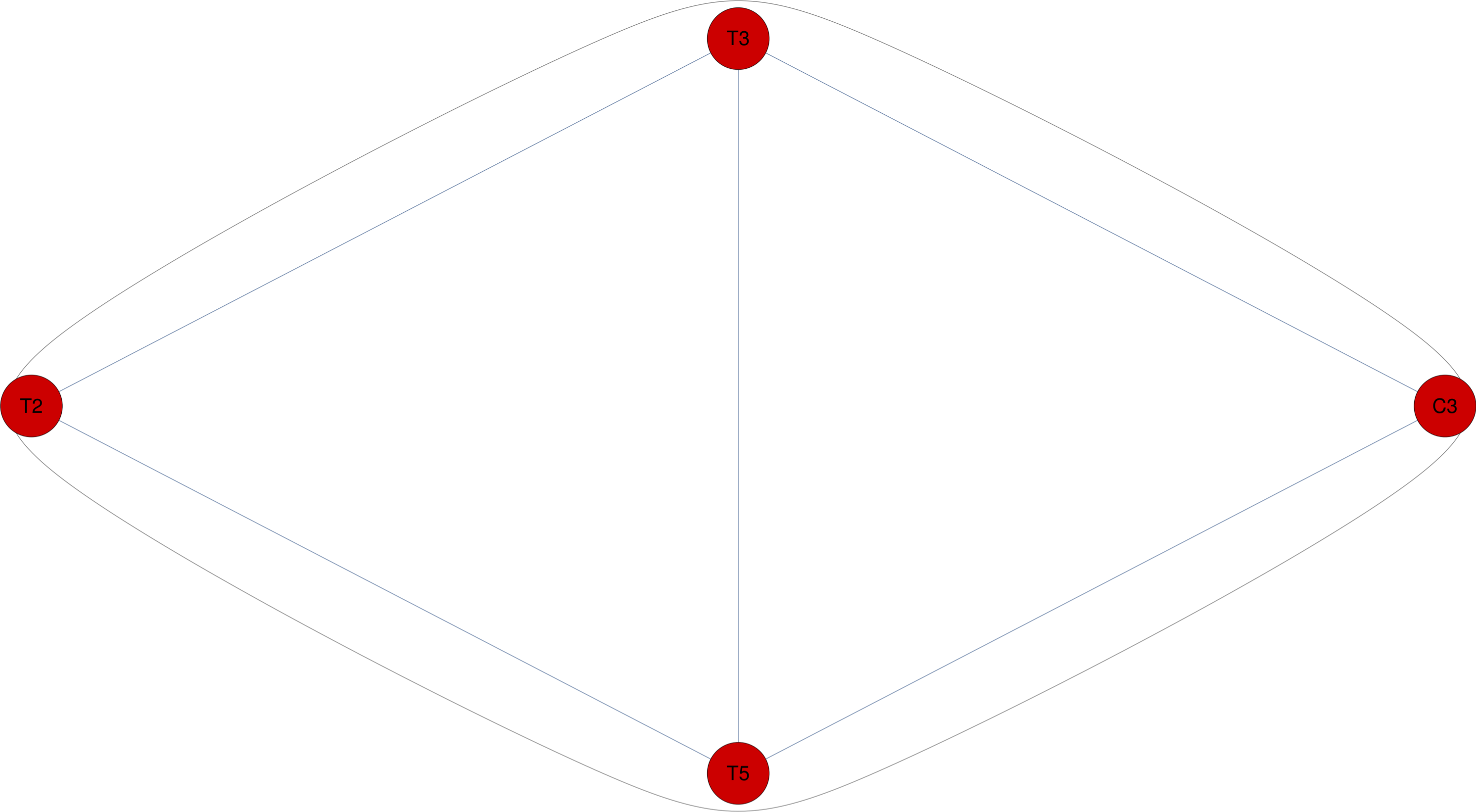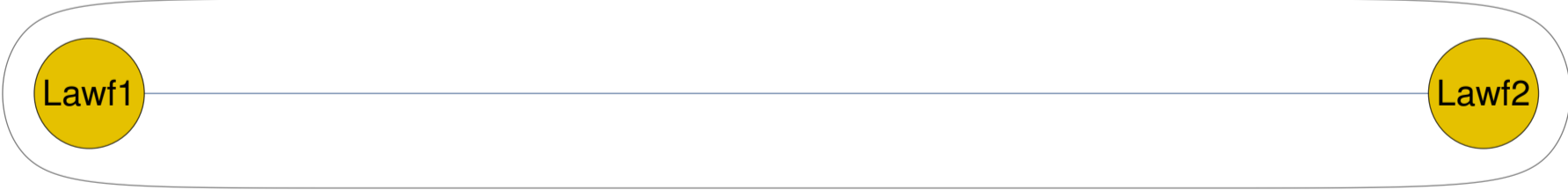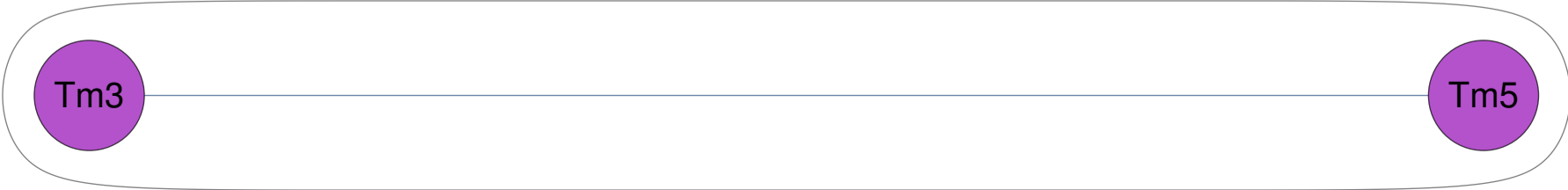

**S8D Fig**

Supplement: S8 Fig — (A) Graph showing community structure of neuron types that appear together at least one time (occurrence = 1). (A) Graph for occurrence = 2. (B). Graph for occurrence = 3 (C). Graph for occurrence = 4 (D). (PDF) [file pone.0227897.s008.pdf]

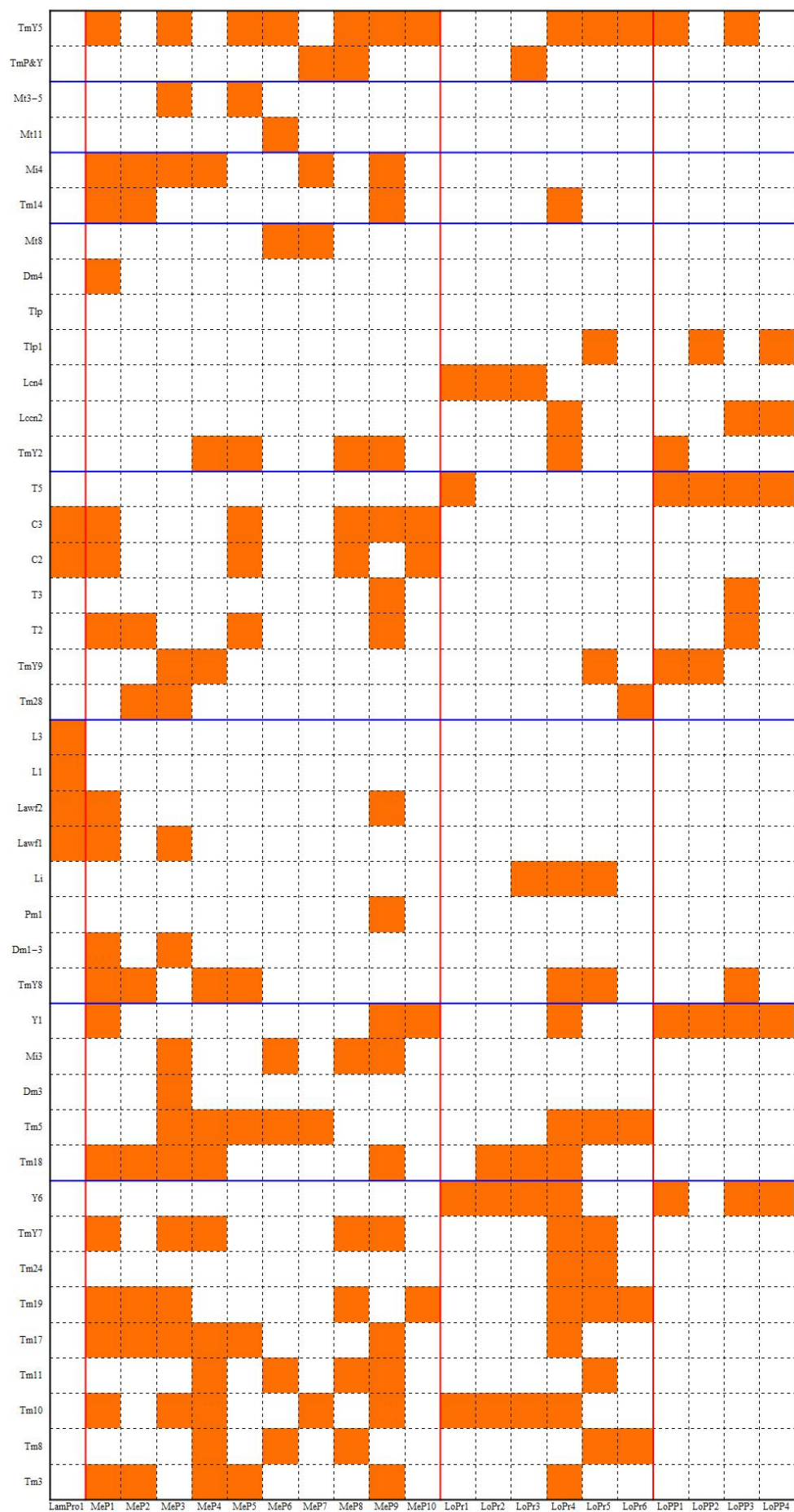

**S9 Fig**

Supplement: S9 Fig — Axonal projections of the neuron types from the 8 communities to the different layers of the optic lobe (layers 1, 1–10, 1–6, 1–4 of the lamina, medulla, lobula and lobula plate neuropils). (PDF) [file pone.0227897.s009.pdf]

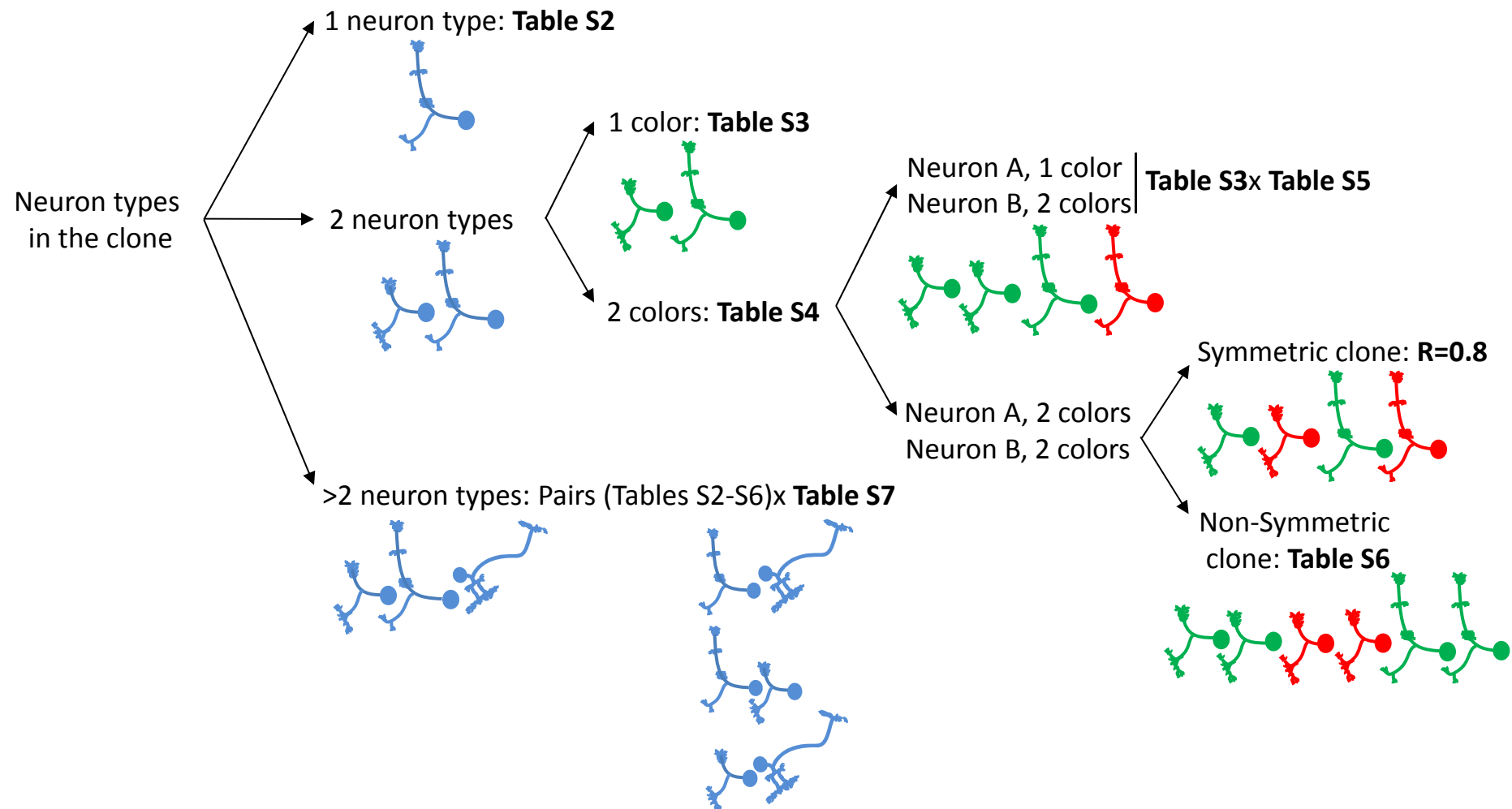

**S10 Fig**

Supplement: S10 Fig — The flow chart shows all the possible combinations of pairs of neurons in our clones and the correction coefficients to be applied from S2–S7 Tables. (PDF) [file pone.0227897.s010.pdf]
