# Supplementary material for: A network approach to analyze neuronal lineage and layer innervation in the Drosophila optic lobes
Source: PLoS One. 2020 Feb 5;15(2):e0227897. doi: 10.1371/journal.pone.0227897 (PMC7001925; doi:10.1371/journal.pone.0227897)
Supplement: S2 Fig — We show the different pairs of neurons within the clone in Fig 3E and calculate the R for each of them according to the S2–S7 Tables. t2-t7 indicate the (S2 to S7) Table applied to each pair. The value 0.72, obtained from S2 Table and the total number of neurons in a clone (S6 Table), multiplies every pair value to obtain the final R for each pair. (PDF) [file pone.0227897.s002.pdf]

3 {{“Tm8”,2,1},{“Tm3”,1,1}, {“Dm3”,1,0}, {“Y1”,1,0}, {“Tm18”,2,0}}

10 pairs: (t2)0.9*(t7)0.8=0.72*

{{"Dm3",0,1}, {"Tm18",0,2}}: (t3) 0.95=0.68
{{"Dm3",0,1}, {"Tm3",2,2}}: (t4)0.95*(t5)0.75=0.51
{{"Dm3",0,1}, {"Tm8",1,2}}: (t4)0.95*(t5)0.75=0.51
{{"Dm3",0,1}, {"Y1",0,1}}: (t3)0.95=0.68
{{"Tm18",0,2}, {"Tm3",2,2}}: (t4)0.7*(t5)0.75=0.38
{{"Tm18",0,2}, {"Tm8",1,2}}: (t4)0.7*(t5)0.75=0.38
{{"Tm18",0,2}, {"Y1",0,1}}: (t3)0.95=0.68
{{"Tm3",2,2}, {"Y1",0,1}}: (t4)0.95*(t5)0.75=0.51
{{"Tm8",1,2}, {"Y1",0,1}}: (t4)0.95*(t5)0.75=0.51
{{"Tm3",1,2}, {"Tm8",1,2}}: (t6)0.6=0.43

S2 Fig
