# Supplementary material for: A network approach to analyze neuronal lineage and layer innervation in the Drosophila optic lobes
Source: PLoS One. 2020 Feb 5;15(2):e0227897. doi: 10.1371/journal.pone.0227897 (PMC7001925; doi:10.1371/journal.pone.0227897)
Supplement: S1 Appendix — Distribution of R for all pairs of cells, number of cell types in each clone, distribution versus number of clones, mean, standard deviation and quartiles are shown. (PDF) [file pone.0227897.s011.pdf]

Appendix 1: Statistical analysis of R.

1.1 R distribution for all pairs of cells:

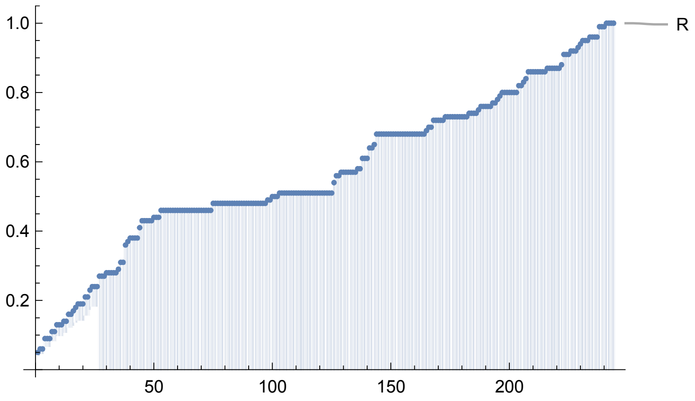

1.2 Number of cell types in each clon:
1 cell type: 134 clones
2 cell types: 110 clones
3 cell types: 44 clones
4 cell types: 12 clones
5 cell types: 8 clones

NB. The difference up to 350 corresponds to cell clones with one cell type and unidentified neurons.

1.3 R distribution vs. number of clones:

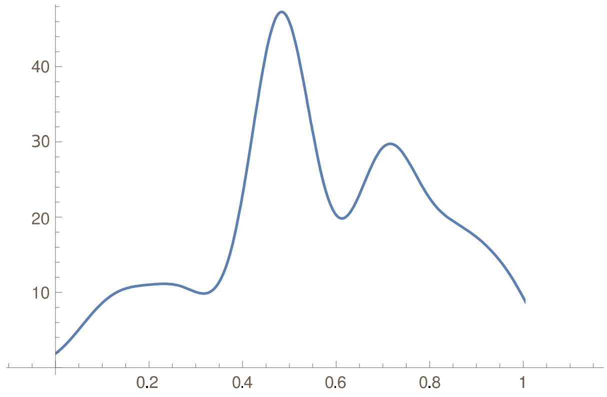

This graph shows that most pairs have an R between 0.4-0.6, with another peak in the interval 0.65-0.75, where the maximum modularity stands.

1.4 Other statistical parameters are:
Mean: 0.573484
Standard deviation: 0.23072
Quartiles: 46,51,74
