# Supplementary material for: A network approach to analyze neuronal lineage and layer innervation in the Drosophila optic lobes
Source: PLoS One. 2020 Feb 5;15(2):e0227897. doi: 10.1371/journal.pone.0227897 (PMC7001925; doi:10.1371/journal.pone.0227897)
Supplement: S2 Table — (PDF) [file pone.0227897.s013.pdf]

Table 2: Clones with one cell type

Quality	Correction coefficient
0	0.10
1	0.25
2	0.60
3	0.90
4	1.00

S2 Table
