# Supplementary material for: A network approach to analyze neuronal lineage and layer innervation in the Drosophila optic lobes
Source: PLoS One. 2020 Feb 5;15(2):e0227897. doi: 10.1371/journal.pone.0227897 (PMC7001925; doi:10.1371/journal.pone.0227897)
Supplement: S3 Table — (PDF) [file pone.0227897.s014.pdf]

Table 3: Clones with two cell types of the same color

Total neurons		cc
(1-4,0)	(1-4,0)	0.95
(5-10,0)	(1-10,0)	0.85
(11-20,0)	(1-20,0)	0.75
(>20,0)	(x,0)	0.50

S3 Table
