# Supplementary material for: A network approach to analyze neuronal lineage and layer innervation in the Drosophila optic lobes
Source: PLoS One. 2020 Feb 5;15(2):e0227897. doi: 10.1371/journal.pone.0227897 (PMC7001925; doi:10.1371/journal.pone.0227897)
Supplement: S4 Table — (PDF) [file pone.0227897.s015.pdf]

Table 4: Clones with two cell types of different color

Total neurons		cc
(1,0)	(0,1)	0.95
(2-4, 0)	(0,1-4)	0.70
(5-10,0)	(0,1-10)	0.55
(11-20, 0)	(0,1-20)	0.40
(>20,0)	(0,x)	0.25

S4 Table
