# Supplementary material for: A network approach to analyze neuronal lineage and layer innervation in the Drosophila optic lobes
Source: PLoS One. 2020 Feb 5;15(2):e0227897. doi: 10.1371/journal.pone.0227897 (PMC7001925; doi:10.1371/journal.pone.0227897)
Supplement: S5 Table — (PDF) [file pone.0227897.s016.pdf]

Table 5: Clones with two neuron types of different color

Total bicolor neurons	Correction coefficient
2-4	0.75
5-10	0.50
11-20	0.35
>20	0.20

S5 Table
