# Supplementary material for: A network approach to analyze neuronal lineage and layer innervation in the Drosophila optic lobes
Source: PLoS One. 2020 Feb 5;15(2):e0227897. doi: 10.1371/journal.pone.0227897 (PMC7001925; doi:10.1371/journal.pone.0227897)
Supplement: S6 Table — (PDF) [file pone.0227897.s017.pdf]

Table 6: Clones with two neuron types of two colors

Bicolor neurons	Correction coefficient
2-4	0.6
5-10	0.35
11-20	0.20
>20	0.05

S6 Table
