# Supplementary material for: A network approach to analyze neuronal lineage and layer innervation in the Drosophila optic lobes
Source: PLoS One. 2020 Feb 5;15(2):e0227897. doi: 10.1371/journal.pone.0227897 (PMC7001925; doi:10.1371/journal.pone.0227897)
Supplement: S7 Table — (PDF) [file pone.0227897.s018.pdf]

Table 7: Clones with more than two neuron types

	cc	
Total neuron types in the clone	<30	≥30
2	1.00	1.00
3	0.90	0.80
4	0.85	0.50
5	0.80	0.30

S7 Table
